# DFT and Mass Spectrometry Study of (−)ESI-Induced Fragmentation of the Highly Toxic Rodenticide Tetramethylenedisulfotetramine

**DOI:** 10.1021/acsomega.6c00837

**Published:** 2026-05-08

**Authors:** Jonathan L. Gertner, Jacob Cook, Brett Mayer, Janel E. Owens, Amanda Morgenstern

**Affiliations:** Department of Chemistry & Biochemistry, 14676 University of Colorado Colorado Springs, Colorado Springs, Colorado 80918, United States

## Abstract

The toxic heteroadamantane rodenticide, tetramethylenedisulfotetramine (TETS, C_4_H_8_N_4_O_4_S_2_; 240 g/mol), undergoes an unusual in-source condensation reaction and ionization process when ionized by (−)­electrospray ionization (ESI) under acidic conditions. TETS either condenses to hexamethylenetrisulfohexamine (HEXS, C_6_H_12_N_6_O_6_S_3_; 360 g/mol), which is protonated in the acidic mobile phase prior to losing a methylene group, or [M + H – CH_2_]^−^ where M = HEXS, to form the precursor ion at *m*/*z* 347, or directly forms the precursor ion *m/*z 227, [M + H – CH_2_]^−^ where M = TETS. Experimental and computational data suggest that the precursor ions are formed in the microdroplet rather than through gas-phase collision-induced dissociation. Here, potential mechanisms of the ionization pathways of TETS, HEXS, and hexamethylenetetramine (HMT), a related heteroadamantane with no sulfonyl moieties, within the ESI source were investigated with density functional theory. The quantum theory of atoms in molecules, interacting quantum atoms, and fragment energy methods were applied to rationalize activation barriers. Results suggest that the proposed pathways could be achieved on the ESI microdroplet time scale (∼10 μs) for TETS and HEXS, which had similar activation energies, but not for HMT. LC/MS/MS experiments were also performed to consider the effect of instrument parameters on fragmentation. The capillary voltage was the only parameter tested that statistically significantly affected the formation of *m*/*z* 347 relative to *m*/*z* 227, suggesting that capillary voltage is an important contributor to the condensation reaction rather than ionization rates.

## Graphical abstract

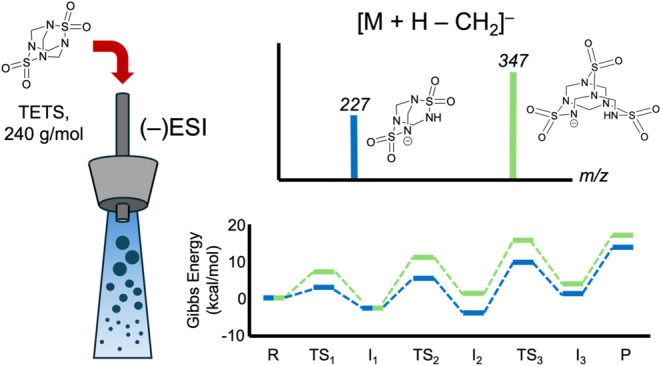

## Introduction

Qualitatively, mass spectrometry ionization sources have been categorized as ‘hard’, resulting in ions that have high average internal energy that fragment easily, or ‘soft’, where the resulting ions have low internal energy, and the mass spectrum is dominated by the presence of a pseudomolecular ion of [M + H]^+^ or [M – H]^−^.^1^ The use of soft ionization techniques, like electrospray ionization (ESI), especially when coupled with high-performance liquid chromatography (HPLC), has enabled tremendous scientific advancement in many fields (metabolomics, toxicology, pharmaceutical development, environmental analyses, to name a few). While ESI is a ‘soft’ ionization technique, ESI-produced spectra can be difficult to interpret because of the presence of cation-adducts,^2^ multimers,^3^ and product ions^2^ resulting from in-source fragmentation (ISF). In the latter case, ISF occurs because of harsh parameters of the source (i.e., voltage settings, capillary temperature, solvent composition and pH, type and pressure of collision gas) that alter the internal energy of the ionized molecule.^4^

The addition of mobile phase modifiers, especially when HPLC is coupled to ESI, has long been thought to support the enhanced formation of [M + H]^+^ ions or [M – H]^−^ ions during the ESI process. In important experiments starting in the mid-1990s, several research groups observed what was ultimately called ‘wrong-way-round’ ESI. These experiments resulted in the formation of high intensity negatively charged ions under acidic conditions,^5–8^ and the resulting mass spectra produced via ESI do not reflect equilibrium concentrations of ions in solution.^6^

Further complicating spectra interpretation, in-droplet reactions can occur during ESI in addition to collision-induced dissociation (CID) ISF. Microdroplets, such as those produced during ESI, have been shown to have acceleration factors greater than 10^4^ for a variety of organic reactions including Hanzstch, Fischer Indole, Biginelli, and Mannich-type reactions.^9–12^ The exact mechanism of microdroplet rate acceleration is unclear, but it has been suggested that rate enhancements for ESI in-droplet reactions are due to some combination of concentration and pH extremes due to evaporation, partial solvation at the liquid–gas interface, and ion double-layer electric field effects.^13,14^ Unfortunately, it is often difficult to distinguish between gas phase (CID ISF) and solvated (microdroplet) reactions during ESI. Other sources, like DART, may allow researchers to isolate gas-phase versus microdroplet reactions.^15^

There are reports in the literature in which validated quantitative analytical methods have been presented for several analytes or classes of analytes that experience ISF of the selected precursor ion prior to multiple reaction monitoring (MRM) analysis. Pesticides,^16,17^ including the highly toxic rodenticide tetramethylenedisulfotetramine (TETS, C_4_H_8_N_4_O_4_S_2_),^18^ must be detected at low analytical limits because of associated toxicity, and hence accuracy and reproducibility of quantitative methods is essential. TETS is a sulfonyl-containing thia and aza-substituted heteroadamantane formed by facile condensation of formaldehyde and sulfamide^19^ characterized by extremely high toxicity^20^ and high chemical stability.^21^ While TETS is typically analyzed by gas chromatography–mass spectrometry (GC/MS),^22^ there are only two reports, to our knowledge, in which TETS was analyzed by LC/MS/MS using (−)­ESI.^18,23^ During the LC-(−)­ESI-MS/MS analyses of TETS, the in-source condensation of TETS to hexamethylenetrisulfohexamine (HEXS, C_6_H_12_N_6_O_6_S_3_), a pseudodimer of TETS, was observed. HEXS then underwent ionization with a complete absence of any intact pseudomolecular ions that are typically expected in (−)­ESI (i.e., [M – H]^−^). The observed *m*/*z* 347 precursor ion peak was assumed to be formed through the loss of a methylene group in protonated HEXS. Given the highly unusual nature of this ionization via (−)­ESI, the precursor and product ions were monitored via high resolution and high-mass accuracy MS via an Orbitrap FT mass spectrometer (Thermo Fisher Scientific) in that original report.^18^ While the condensation of HEXS from TETS in the presence of acidic and heated environments is well established^24^ and recent reports of caged amine synthesis via condensation reactions in droplets exist,^25^ the analyte condensation with subsequent ISF represents a rare phenomenon that warrants further investigation.

There are two additional reasons the formation of a *m*/*z* 347 peak from HEXS is highly unusual. First, most reports of ISF in the literature involve the breakage of a single bond. Previous work suggested the *m*/*z* 347 peak was formed from the breakage of two neighboring C–N bonds within a ring. We are not aware of any other reports of ISF resulting in loss of a methylene group within a ring structure such as occurs for HEXS. Second, the unusual in-source fragmentation of HEXS was only observed with negative ion mode ESI but the LC/MS/MS solvents both included formic acid. Based on previous experimental work^18^ and data presented here, we propose the following mechanism for TETS resulting in [M + H – CH_2_]^−^ precursor ions from (−)­ESI (Scheme 1). During ESI, some of the TETS forms HEXS-H^+^ due to the presence of formic acid and heat, while other TETS molecules are simply protonated (TETS-H^+^). TETS-H^+^ and HEXS-H^+^ ions then undergo in-droplet reactions resulting in the loss of a methylene group as formaldehyde. As the droplet evaporates, but possibly after the loss of the methylene group, hydroxide or formate ions begin to interact with this compound, leading to the observed [M + H – CH_2_]^−^ precursor ion. During MS/MS, the [M + H – CH_2_]^−^ precursor ions from HEXS are detected via MRM as *m*/*z* 347 → *m*/*z* 227 and the [M + H – CH_2_]^−^ precursor ions from TETS are detected via MRM as *m*/*z* 227 → *m*/*z* 134 in this work.

**Scheme 1:**
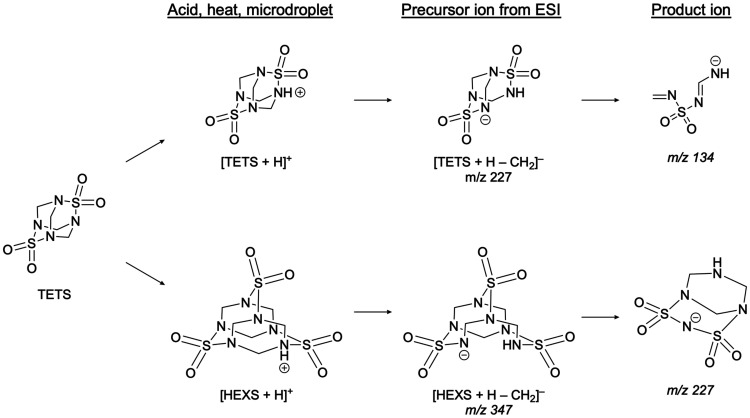
Proposed Mechanism for the Formation of (−)­ESI-LC/MS/MS *m*/*z* 134 and *m*/*z* 227 Fragments as Observed Product Ions from Precursor Ions *m*/*z* 227 and *m*/*z* 347, Respectively^a^

a The proposed structures of the ions resulting from analyses completed via high resolution and high mass accuracy mass spectrometry using an Orbitrap FT mass spectrometer in previous work with an error of less than 4 ppm.^18^

We investigate the plausibility of this mechanism through the calculation of activation barriers starting with the protonated compounds and ending with the [M + H – CH_2_]^−^ precursor ions for TETS, HEXS, and hexamethylenetetramine (HMT, C_6_H_12_N_4_). HMT was included in this study as a control C–N heteroadamantane devoid of sulfonyl moieties. To rationalize the calculated activation energies, the stabilities of energy minima along reaction pathways were analyzed via the quantum theory of atoms in molecules (QTAIM), interacting quantum atoms (IQA), and fragment energy analysis methods. These *in silico* results were compared to ESI time scales to evaluate the likelihood of microdroplet fragmentation of HEXS and TETS to form the observed *m*/*z* 347 and *m*/*z* 227 precursor ions. GC/MS, LC/MS/MS, and ^1^H NMR experiments were also performed to validate computational results and determine the effect of LC/MS/MS instrument parameters on precursor ion formation.

## Materials and Methods

### Chemicals and Reagents

Tetramethylenedisulfotetramine (TETS) was kindly provided by Lawrence Livermore National Laboratory (LLNL) and synthesized as previously described.^18^ HPLC-grade acetone, Optima LC/MS-grade acetonitrile, Optima LC/MS-grade formic acid, and hexamethylenetetramine (HMT) were from Fisher Scientific (Fair Lawn, NJ). Organic-free DDI 18 MΩ water was produced using a Millipore filtration system (St. Louis, MO).

A stock solution of TETS was prepared by dissolving 2 mg in 1 mL of acetone. Stock solutions of HMT were prepared by dissolving 25 mg in 25 mL of acetone. Stock solutions were stored at 4 °C until they were diluted to prepare analytical standards. Analytical standards of TETS (48 ng/μL) and HMT (2 ng/μL) in acetone were subsequently prepared for GC/MS and LC/MS/MS analyses. Given the highly toxic nature of TETS, analytical stock solutions and standards were prepared in a chemical fume hood while wearing full personal protective equipment. Any laboratory consumables or solvent waste streams were separated from normal laboratory processes to be expressly double-bagged and specially labeled for waste processing.

### Instrument Conditions

#### GC/MS

To confirm the purity of the TETS standard provided by LLNL colleagues, the prepared standard in acetone was analyzed via full-scan GC/MS using an HP 6890 GC coupled with a 5973N MSD. One μL injections using an autosampler were made onto a DB-5 ms column (25 m × 0.200 m i.d. × 0.33 μm film thickness) with hydrogen as a carrier gas at 0.7 mL/min. The inlet was maintained at 250 °C and operated in split/splitless mode. Upon injection, the column oven was 60 °C (hold for 3.00 min) with a subsequent ramp at 30.0 °C/min to 305 °C (hold for 5.00 min) for a total run time of 16.17 min. TETS eluted at 10.993 min (Figure S1). The auxiliary transfer line was at 280 °C while the MSD source was at 230 °C and the quadrupoles at 150 °C. After a 6.0 min solvent delay, TETS was analyzed in full-scan mode (*m*/*z* 50.00–450.00) with 3.58 scans per second with a relative voltage increase of 400 V to the electron multiplier above tune conditions.

A TETS standard at 20 ng/μL was also incubated with formic acid for 1 week at room temperature (25 °C) and reanalyzed via GC/MS to confirm that the presence of formic acid alone would not cause the formation of HEXS. Supporting chromatograms are provided in Figure S2.

#### LC/MS/MS

Analytical standards of TETS were analyzed via a Shimadzu Prominence series HPLC coupled to a Shimadzu LCMS-8030 LC/MS/MS system. Two μL injections of the analytical standard of TETS (48 ng/μL) were isolated on a Waters Symmetry300 C4 column (100 mm × 2.1 mm i.d., 3.5 μm particle size; Milford, MA) using isocratic conditions of 5/95 (v/v) solvent A and solvent B held at a constant flow rate of 0.3000 mL/min. Solvent A was organic-free DDI 18 MΩ water with 0.1% formic acid, and mobile phase B was acetonitrile with 0.1% formic acid, with a total run time of 1.50 min, with TETS eluting at 1.10 min as TETS (*m*/*z* 227) and HEXS (*m*/*z* 347) (Figure S3). The analytical standard of TETS was held at 15 °C in the autosampler between injections. The column temperature was 40 °C.

TETS was analyzed in negative-ion mode ESI and operated in selected ion monitoring (SIM) mode for *m*/*z* 347 (HEXS) and *m*/*z* 227 (TETS) and multiple reaction monitoring mode (MRM) for *m/*z 347 > 227 (HEXS) and *m/*z 227 > 134 (TETS), where collision energy was set to 5.0 V for both transitions. Dwell times for ions ranged between 24.0 and 80.0 ms, depending on the experiment. The desolvation line temperature was maintained at 250 °C for all experiments. The following parameters were adjusted to three different settings for a Box-Behnken model for three parameters in two different experimental models: capillary voltage was −1.5, −3.0, and −4.5 kV (experiment 1); nebulizing gas flow as 0.5, 1.5, and 3.0 L/min (experiment 2); drying gas was 3 L/min, 11 L/min, and 20 L/min (experiment 1) or 0 L/min, 10 L/min, and 20 L/min (experiment 2); and the heat block was held at 50 °C, 150 °C, and 300 °C (experiments 1 and 2).

#### ^1^H NMR

The ^1^H NMR spectrum of TETS (∼2 mg, 8 μmol) in CD_3_CN was produced using a Bruker 400 MHz NMR with resulting chemical shifts (δ, ppm) relative to TMS: 5.5 (8H, s; Figure S5), which compared to a previously published literature report.^26^ Formic acid (0.8 μL; 20 μmol) was incubated with TETS at ambient temperature (300 K) in the NMR. Resulting peaks for formic acid were observed at 8.0 ppm (1H, s) within this spectrum along with the presence of the hydroxyl proton at 9.5 ppm (s, broad OH), and an amine at 2.1 (s, broad NH from protonation of TETS; Figure S5). For comparison, HMT (∼17 mg; 120 μmol in CD_3_CN) produced a singlet at 4.7 ppm (12 H; Figure S6). Treatment with formic acid (5 μL; 133 μmol) produced no evidence of an amine peak from protonation of HMT. Only formic acid (8.1 ppm) and hydroxyl (10.0 ppm) peaks were observed along with HMT at 4.7 ppm (Figure S6).

### Theoretical Calculations

All DFT calculations were run with the Amsterdam Density Functional (ADF) package of the Amsterdam Modeling Suite (AMS2023.104).^27,28^ Initial geometry optimizations of neutral and protonated compounds were performed with both PBE and B3LYP functionals utilizing an all-electron triple-ζ double polarization (TZ2P) and triple-ζ polarization (TZP) basis set, respectively. Each of these methods was applied using COSMO implicit solvation with parameters for both water (ε = 78.39) and acetonitrile (ε = 37.5) to simulate possible environments during ESI in addition to gas phase calculations. Due to qualitative agreement between resulting geometries, ρ­(r) values at bond critical points (CP), and fragment bonding energies (BE) from these methods when comparing molecular trends, only B3LYP/TZP results with water are shown here (see Table S1 for other method results). For interacting quantum atoms (IQA) analysis, numerical integration accuracy was increased from the default value to the “very good” Becke 4 integration accuracy (estimated bonding energy accuracy Δ*E* of <0.0005 mHartree),^29^ as integration accuracy has a large influence on IQA values. Optimized geometries were verified to have no imaginary frequencies to ensure stable structures.

Stabilities of each compound were analyzed using QTAIM, IQA, and fragment BE methods. Within QTAIM, the values of ρ­(*r*) at C–N bond CPs and bond path lengths were evaluated to compare bond strengths between and within compounds. IQA was used to decompose the total interaction energies of each system into pairwise interactions of individual atoms. Fragment BEs were calculated for reactant states where the methylene group that was lost during the reaction was defined as one fragment and the rest of the molecule was defined as the second fragment. For solvated results, both the total compound and each fragment were solvated. Fragment BEs then provided the change in energy of the total compound from the two isolated fragments (with same geometry as in the total compound). Some intermediates were also analyzed with fragment BEs, but in this case, the protonated formaldehyde was defined as one fragment, and the rest of the molecule was defined as the second fragment. As an example, the BE of the methylene group in TETS-H^+^ is calculated with

1 $$\mathrm{BE}\;=E_{\mathrm{TET}{S-H}^{+}}-\left(E_{CH_{2}^{+2}}\;+\;E_{\mathrm{res}t^{-}}\right)$$

Possible fragmentation pathways during ESI were determined for HMT-H^+^, TETS-H^+^, and HEXS-H^+^. A general mechanism was first devised (Scheme S1), consisting of five steps. During modeling, variations on this proposed mechanism were explored and the lowest energy pathways are included in Results and Discussion. Transition states were calculated for each step and verified as having one imaginary frequency. Subsequent intrinsic reaction coordinates following the vibrational mode corresponding to the imaginary frequency were performed to ensure each transition state corresponded to the appropriate reactant and product states. All electron B3LYP/TZP calculations were performed for all pathways. B3LYP was chosen to balance calculation cost and accuracy. B3LYP has been shown to produce average errors of 5.96 kcal/mol for barrier heights in a large benchmarking study^30^ and similar errors for dissociation threshold energies used to study CID ISF.^31^ However, the work by Asakawa et al., showed that B3LYP consistently underestimated energies, which will allow most errors to cancel when comparing compounds in this work.^31^ For condensed phase calculations, COSMO solvation was used with water as the solvent (ε = 78.39), and two explicit water molecules were included in the model, as has been done in other work modeling fragmentation pathways.^32^ Some steps, especially the final step forming the (−) precursor ion, were found to be endothermic with no discernible transition state, as has been found in other mass spectrometry fragmentation studies.^33–36^ Final reported Gibbs energies are reported for 300 °C, in line with LC/MS/MS experimental conditions.

## Results and Discussion

### Confirmation of TETS by GC/MS

Before analysis of the TETS analytical standard by LC/MS/MS was completed, the analytical standard was analyzed by GC/MS to verify that there was no presence of HEXS within the analytical standard. Previous work had demonstrated that a very small amount of HEXS could be produced via synthesis of TETS with sulfamide and trioxane,^18^ but collected data here suggested that no HEXS was present within our 48 ng/μL standard prepared in acetone, even when incubated with formic acid for 1 week and exposed to high temperatures (up to 305 °C) during GC/MS (Figure S2).

### LC-(−)­ESI-MS/MS

The addition of volatile acids to mobile phases with resulting high intensity negative ions has previously been explored.^5–8^ Gatlin and Tureček first observed that the relative acidity of a droplet solution can be substantially higher than the bulk mobile phase with a 10^3^ to 10^4^ fold increase in hydronium ion concentration, and that there was a surface layer in spherical microdroplets formed in (+)­ESI experiments that had a high local acidity where dissociation reactions could occur.^5^ Hiraoka et al., explored further the effects of mobile phase modifiers in the ionization of small amino acids by both positive and negative ESI. Interestingly, [M – H]^−^ ions could result upon the deprotonation of M by the conjugate base of the weak acid added as a modifier. Additionally, the appearance of hydroxide ions was noteworthy in these acidic mobile phases because these OH^–^ ions appeared from the electrochemical reduction of water at the metal/liquid interface of the capillary needle of the ESI source: O_2_ + 2H_2_O + 4e^–^ ⇌ 4OH^–^ with *E*_0_ = 0.4 *V*.^6^ Wu et al., similarly observed that addition of weak volatile acids significantly increased response under (−)­ESI. These free protons from the acid modifiers could facilitate reduction at the (−)­ESI needle to make it easier for the formed spray droplets to carry the excess negative charge that is accumulating on the surface of the droplet. These droplets are then selected according to the polarity of the potential applied within ESI.^8^

Thus, the addition of formic acid in the mobile phase and the temperature of the ESI source suggest that either the high negative charge on the droplet and/or specific conditions spurred by the addition of formic acid in the presence of heat for sufficient time are crucial for the formation of HEXS (GC peak of *m*/*z* 360) to HEXS-H^+^ from TETS (GC peak of *m*/*z* 240) prior to losing a methylene bridge (−CH_2_−) to form the negative precursor ion of *m*/*z* 347 within ESI. The formation of this precursor ion and subsequent product ions were investigated in our prior work using (−)­ESI coupled to an Orbitrap mass spectrometer, with reported mass error values of <4 ppm for precursor and most product ions observed.^18^ Moreover, the presence of the sulfonyl moieties also appears to be critical for the formation of the [M + H – CH_2_]^−^ ion, as our control heteroadamantane, HMT, formed no negative ions when analyzed via LC/MS, although [M + H]^+^ ions were readily observed with (+)­ESI (Figure S4).

We further investigated the importance of droplet formation on ionization efficiency (as measured by resulting peak area from the LC/MS and LC/MS/MS chromatograms) and preferential formation of [HEXS + H – CH_2_]^−^, or *m*/*z* 347, over the formation of protonated TETS with methylene bridge loss, [TETS + H – CH_2_]^−^, or *m*/*z* 227, using Box-Behnken designs for three factors: capillary voltage, drying gas flow rate, and heat block temperature. While several instrumental parameters can be altered to optimize ESI analyses,^37^ past reports by others suggested that altering temperature profiles^11,38^ and nebulizing gas flow rates,^39^ would be important factors to consider when investigating ESI instrumental parameter settings on improving efficiency of reactions that occur in the source. Recent work by Hongo et al., highlighted the impact of corona-discharge effects with increasing ESI capillary needle voltage on forming unwanted in-source reaction products during metabolome profile studies.^40^

### Varying Experimental Parameters

The Box–Behnken design was used to determine the main and interactive effects of parameters on the formation of *m*/*z* 347 and *m*/*z* 227 ions in SIM mode and as precursor ions in MRM (see Figure S3 for an example chromatogram that was integrated to report peak area for statistical analyses). A total of 45 trials were completed with running each parameter combination in triplicate, with all peak areas recorded for all trials. Combinations of capillary voltage (−1.5 kV, −3.0 kV, −4.5 kV), drying gas flow rate (3 L/min, 11 L/min, 20 L/min), and ESI heat block temperature (50 °C, 150 °C, and 300 °C) were analyzed first. Single-factor ANOVA was used to assess the statistical significance between main and interactive effects (Table S2).

A higher voltage of −4.5 kV produced statistically significantly higher peak areas on resulting integrated chromatograms when analyzed in SIM mode (Table S2), an effect that was also observed for all MRM analyses. The ratio of peak areas of *m*/*z* 347/*m*/*z* 227 (or HEXS/TETS via SIM) was also statistically significantly different for the three voltages investigated, with −1.5 kV producing a lower response compared to the capillary voltage settings of −3.0 kV and −4.5 kV, which had no statistically significant difference between them (*P* = 0.0786 by Student’s *t*-test for two-tailed analyses). These results suggest that increasing ESI voltage preferentially increased HEXS ion formation over TETS by factors of 1.12 at −1.5 kV, 1.25 at −3.0 kV, and 1.33 at −4.5 kV. These observations were confirmed for MRM analyses as well, where the resulting peak areas at −1.5 kV were statistically significantly lower than the higher voltage settings (−3.0 and −4.5 kV). There was no statistical difference between −3.0 kV and −4.5 kV for the ratio HEXS/TETS (*P* = 0.1221 by Student’s *t*-test, two-tailed), suggesting that ionization efficiency was affected for both ions. See Supporting Information, Section 6 for a brief discussion on ionization efficiency.

Similar statistical analyses using single-factor ANOVA were completed to assess the main effect of drying gas flow rate, which flows from the MS transfer line orifice orthogonally toward the ESI needle in our instrument source, on peak areas in resulting chromatograms. While drying gas flow rates at 11 L/min and 20 L/min produced statistically significantly improved ionization efficiency as indicated by peak areas versus 3 L/min flow, there was no preferential increase in HEXS versus TETS as indicated by area ratio (Table S2), suggesting that there was no preference for formation of HEXS (*m*/*z* 347) over TETS (*m*/*z* 227) with assumed smaller droplet size. This same trend was observed for the effect of heat block temperature. There was no statistically significant difference between 150 and 300 °C on resulting peak areas for HEXS (*m*/*z* 347) and TETS (*m*/*z* 227), though these temperatures showed statistically significantly improved ionization efficiency over 50 °C. Importantly, however, there was no preferential increase in HEXS versus TETS as indicated by area ratios for SIM and MRM analyses (Table S2).

In summary, the formation of the HEXS ion (*m*/*z* 347) and TETS ion (*m*/*z* 227) was improved with higher gas flow rates and higher temperature of the source, which would be expected for any analyte. The preferential formation of HEXS (*m*/*z* 347) over TETS (*m*/*z* 227) was achieved only with higher ESI capillary needle potential, suggesting that this more extreme environment was required for the microdroplet reaction to form the protonated pseudodimer of HEXS from TETS prior to the loss of the methylene bridge. Additional parameters were investigated in a second Box-Behnken design (see Table S3), though no new insights concerning preferential formation of *m*/*z* 347 over *m*/*z* 227 were gained.

### Reaction Pathways

We first explored the effect of nitrogen protonation on the stability of adjacent methylene groups as protonation was originally assumed to occur for the formation of [M + H – CH_2_]^−^ ^18^ and H NMR results from this work showed that TETS can be protonated in formic acid (see Figure S5). For HMT, TETS, and HEXS, all nitrogen atoms are symmetry-equivalent as are the adjacent methylene groups. Thus, one nitrogen atom in each molecule was protonated and subsequent geometry optimizations were performed. Stability of C–N bonds in the condensed phase are compared in Figure 1 for each neutral and protonated compound using the values of ρ­(*r*) at bond CPs (Panel B), bond path lengths (Panel C), and IQA energies (Panel D). As expected, protonation destabilized the C–N bonds in all cases. Across all three molecules, the average percent decrease in bond CP ρ­(*r*) was 14.0%, the average increase in bond path length was 4.1%, and the average increase in IQA bond energy was 11.1% after protonation. The C–N bond in HEXS had the most significant destabilization, which was especially pronounced with the change in IQA energy of 49.7 kcal/mol (15.5%). Based on these results, protonation of a nitrogen atom is assumed to facilitate the loss of a methylene group during (−)­ESI in the acidic medium.

**Figure 1:**
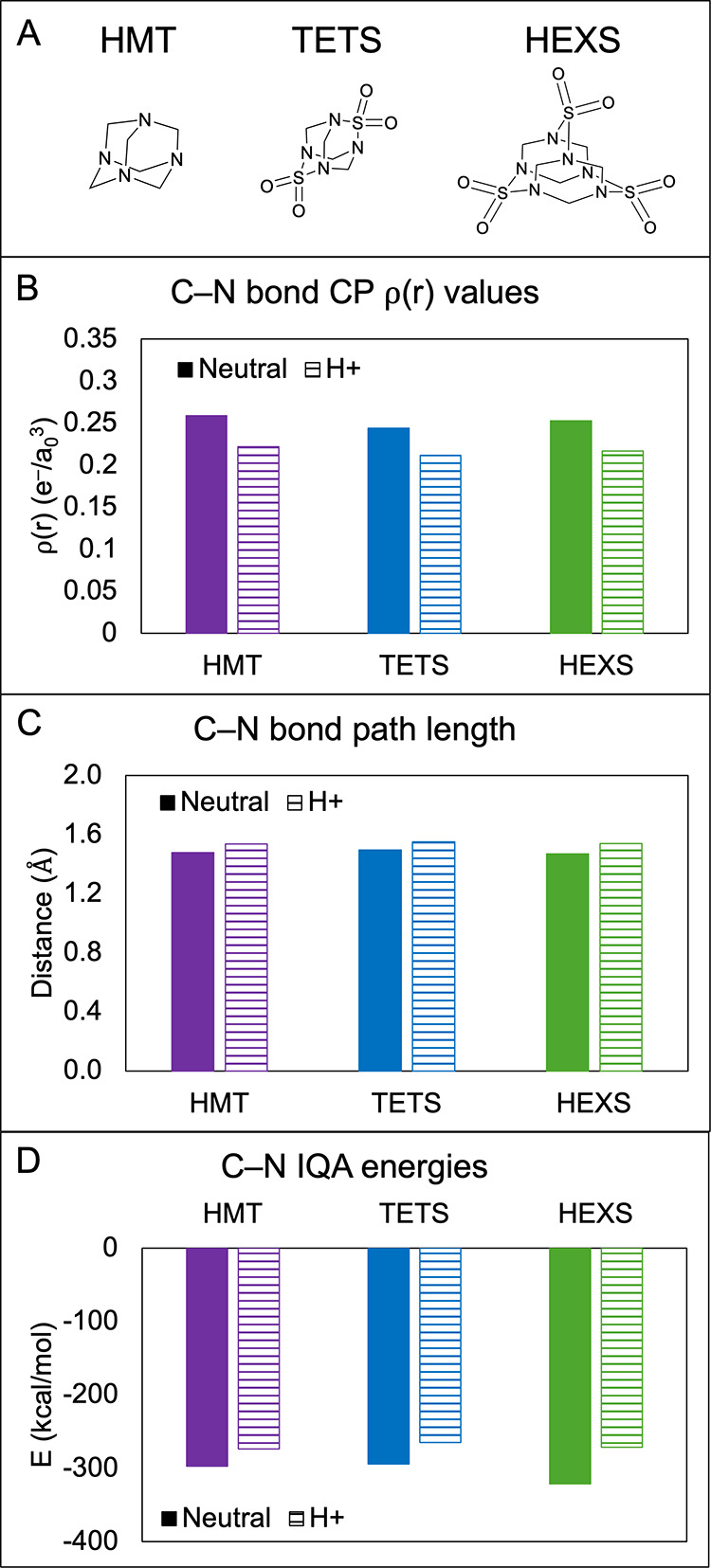
Effect of nitrogen atom protonation on adjacent methylene group stability for HMT, TETS, and HEXS. (A) Neutral molecule structures, (B) bond CP ρ­(*r*) values for the C–NH^+^ bond, (C) C–NH^+^ bond path length, and (D) C–NH^+^ IQA bond energies.

Next, the solvated environment for the reaction pathways starting from protonated compounds was chosen. While the stock TETS solution was formed in acetone and the mobile phase contained water, formic acid, and acetonitrile, water was chosen as the COSMO solvent and the nucleophile in the calculated mechanism. This choice was based on the higher likelihood of water molecules surrounding the sample than acetonitrile during droplet evaporation, as previous molecular dynamics simulations have shown that during droplet evaporation, acetonitrile molecules move toward the outside of droplets and evaporate before water molecules.^41^ Two explicit water molecules were included in calculations to balance the inclusion of hydrogen bonds vital to reaction progress and computational cost, as has been done in other work.^32^ However, we recognize that a variety of molecules could act as a nucleophile in the microdroplet, including formate ions from the formic acid, and emphasize that water was modeled to represent only one possible nucleophile that is readily available.

Potential mechanisms for the loss of a methylene group from protonated HEXS, TETS, and HMT were explored in the condensed phase (COSMO implicit solvation with water) and in the gas phase. While the mechanism is assumed to occur in the droplet, gas phase pathways were also modeled to compare activation barriers and consider the possibility of CID ISF.

A proposed mechanism (Scheme S1) began with the cage opening cleavage of the C–NH^+^ bond to form a tertiary iminium ion. Hydrolysis of the iminium ion by nucleophilic attack then follows to form a hemiaminal, like a retro-Mannich reaction.^42^ Next, protonation of the hemiaminal amine occurred, which yielded a reduction in stability for the bonded hydroxymethyl group. This acid-catalyzed destabilization prompted the second C–N cleavage of the mechanism through protonolysis, resulting in a formaldehyde leaving group from the hemiaminal. The final reaction step was deprotonation, which could occur via formate or hydroxide ions as they are both readily available in (−)­ESI,^6^ resulting in the [M + H – CH_2_]^−^ experimentally observed precursor ion.

The formation of a tertiary iminium ion with COSMO solvation was found to be plausible for HMT (Intermediate 1 (I_1_), Figure 2A, Scheme b), but it was determined that HEXS and TETS undergo C–NH^+^ bond cleavage more favorably through nucleophilic attack on the carbon atom, as shown in Figure 2A, Scheme a. For all three molecules, a concerted hydrogen transfer process was found to be energetically favorable, resulting in I_2_. Both I_1_ and I_2_ correspond to [M + H + H_2_O], or [M + 19]^+^, with proton rearrangement occurring from I_1_ to I_2_. We utilized LC/MS using (+)­ESI in SIM mode and detected *m*/*z* 259 [TETS + 19]^+^ and *m*/*z* 379 [HEXS + 19]^+^ to confirm the formation of these intermediates (see Figure S8).

**Figure 2:**
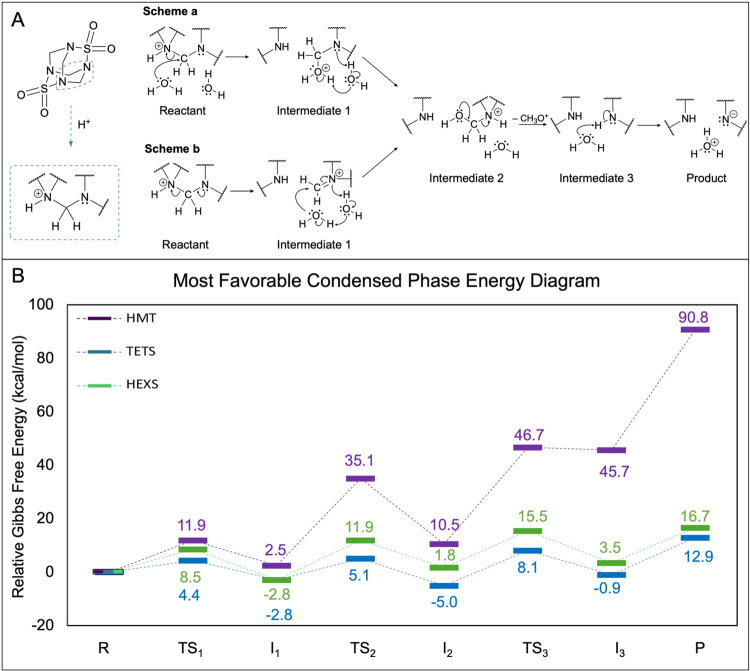
Proposed pathways for formation of [M + H – CH_2_]^−^ with M = HEXS, TETS, or HMT, assuming acidic conditions in aqueous solution. (A) Lowest energy condensed phase pathways found for TETS and HEXS (Scheme a) and HMT (Scheme b) calculated at 300 °C. The dashed box on the left contains the portion of protonated molecules shown for each pathway. (B) Energy diagrams calculated from Scheme a and Scheme b for each molecule.

Rather than the proposed removal of a proton followed by formaldehyde loss, the loss of HCH_2_O^+^ occurred in a concerted step to form I_3_. Attempted hemiaminal deprotonation of the alcohol resulted in positive and negative charged species in proximity, which disallowed a local energy minimum to be found given the system size. Finally, the [M + H – CH_2_]^−^ ions were formed through deprotonation by a water molecule in our calculations. As mentioned earlier, this deprotonation could occur by hydroxide or formate ions during droplet evaporation. Some alternative steps were found to have similar activation barriers, suggesting that multiple pathways can be realized (Scheme S2 and Tables S4 and S5). However, only the lowest energy pathway is discussed here.

Pathways with the lowest activation barriers differed slightly among molecules and in each phase. The lowest-energy condensed-phase pathways for the three molecules from this work are displayed in Figure 2A. HEXS and TETS were found to undergo the Scheme a pathway, while HMT had alternate steps 1 and 2 as shown in Scheme b. Slight variations for the gas-phase pathways of HEXS and TETS are shown in Scheme S3. The HMT gas-phase pathway was identical to the condensed-phase pathway shown here. Activation barriers in the condensed-phase pathway for HEXS and TETS were generally lower than the barriers in the gas-phase pathway, and differences were especially pronounced in the last two steps of the most feasible pathways found. The last step of the mechanism involves the separation of positive and negative ions as modeled here with water as a nucleophile, which resulted in large activation barriers for HEXS and TETS in the gas phase (190.7 and 171.2 kcal/mol, respectively). The activation barriers for HMT were also significantly lower in the condensed phase for reaction steps in the latter half of the proposed pathways (Figure S9).

For the solvated energy diagram shown in Figure 2B, activation barriers increased as the mechanism progressed for HMT, with the largest barrier observed in step 4, where a proton was removed to form the observed negatively charged precursor ion. While water was used as a nucleophile, it is feasible that the latter steps of the proposed mechanism would occur close to the end of droplet evaporation. Since negative charge accumulates on the surface of droplets during (−)­ESI,^43^ these negatively charged ions will interact with a larger volume of the reactants as the droplet evaporates and the surface area of the droplet decreases. In this case, formate ions from the added formic acid and hydroxide ions made via electrochemical reduction of water^6^ would be readily available during (−)­ESI, greatly reducing the activation barrier or making product formation barrierless. Similarly, formate and hydroxide ions might be in contact with I_2_, leading to loss of a proton followed by formaldehyde as originally proposed. The lack of [M + H – CH_2_]^+^ precursor ions during (+)­ESI further suggests the necessity of hydroxide or formate ions for in-source fragmentation. Activation barriers were similar for TETS in steps 3 and 4, while barriers for HEXS were similar in steps 2, 3, and 4. For all compounds, the activation barrier of step 1 was the lowest.

While HEXS had higher condensed phase activation barriers than TETS in the first two steps (by 4.1 and 6.8 kcal/mol, respectively), the step 3 and 4 barriers were nearly identical for these two compounds. Assuming a microdroplet mechanism, HEXS and TETS likely have similar probabilities of generating [M + H – CH_2_]^−^ ions during ESI due to their similar activation barriers. HMT, however, had significantly larger barriers than TETS and HEXS for every step in the condensed phase pathway shown in Figure 2A. The increase in barriers for each step for HMT ranges from 7.5 to 31.2 kcal/mol, which is consistent with experiments that showed HMT does not form a [M + H – CH_2_]^−^ precursor ion during (−)­ESI.

The lifetime of ESI droplets has been estimated to be in the 10–150 μs range.^35,44–47^ Considering a typical ESI heat block temperature of 300 °C, the rate constants for in-droplet fragmentation can be approximated using the Eyring–Polonyi equation,

2 $$k=\kappa \frac{k_{B}T}{h}e^{-\Delta G^{‡}/\mathrm{RT}}$$

where first order reaction kinetics are assumed for each step, and we ignore any dynamical transition state barrier recrossing or competing effects by setting κ = 1. All steps were assumed to only occur in the forward direction for this approximate model. The time for 99% of each reaction step to occur is then estimated with

3 $$t_{99\%}=\frac{-\ln\left(0.01\right)}{k}$$

The values of *t*_99%_ for each step and each compound are shown in Table 1. TETS and HEXS should be able to undergo the proposed mechanism within the microdroplet lifetime, even using a conservative estimate of 10 μs. Considering that microdroplets are known to accelerate reaction rates by 10^1^–10^6^ times, it seems likely that HEXS and TETS could form the [M + H – CH_2_]^−^ precursor ions in-droplet with water, or another molecule, as a nucleophile. If formate or hydroxide ions are available near the end of the proposed mechanism, it is even more likely that this step would be completed in the microdroplet lifetime. For HMT, only step 1 is expected to be able to occur to near completion in the droplet lifetime without taking microdroplet rate acceleration into account, while step 2 and 3 may be able to occur with acceleration. Step 4 for HMT, however, is unlikely to occur in the ESI microdroplet lifetime even with microdroplet rate acceleration. Thus, DFT calculations show that TETS and HEXS are likely able to form [M + H – CH_2_]^−^ ions while HMT is not expected form the [M + H – CH_2_]^−^ ion in (−)­ESI, which matches experimental results.

**Table 1:** Estimated Times for 99% Completion of Reaction Steps from Figure 2B

	step 1 *t*_99%_ (μs)	step 2 *t*_99%_ (μs)	step 3 *t*_99%_ (μs)	step 4 *t*_99%_ (μs)
HMT	1.37 × 10^–2^	1.05 × 10^6^	2.31 × 10^7^	6.08 × 10^10^
TETS	1.84 × 10^–5^	4.22 × 10^–4^	3.99 × 10^–2^	7.44 × 10^–2^
HEXS	6.90 × 10^–4^	1.65 × 10^–1^	6.70 × 10^–2^	4.24 × 10^–2^

### Stability Study

To rationalize the differences in activation barriers between the HEXS, TETS, and HMT pathways, the stabilities of the two C–NH^+^ bonds broken in each compound were examined using values of ρ­(*r*) at bond CPs, fragment BEs, bond path lengths, and IQA energies (Figure 3). The structures preceding C–NH^+^ bond cleavage corresponded to the reactant and I_2_ states. For reactant fragment BEs, the methylene group was assigned a charge state of +2, and the rest of the molecule was assigned a −1 charge, consistent with the hypothetical charges that would exist if both N–CH_2_–NH^+^ bonds broke in the reactant state for each molecule. For I_2_, the protonated formaldehyde was assigned a +1 charge, and the rest of the molecule was assigned a charge state of 0. In Figure 3, larger values of charge density at bond CPs indicate stronger bonds (Panel A), shorter bond path lengths indicate stronger bonds (Panel B), and more negative IQA and fragment energies indicate more stable interactions (Panels C and D).

**Figure 3:**
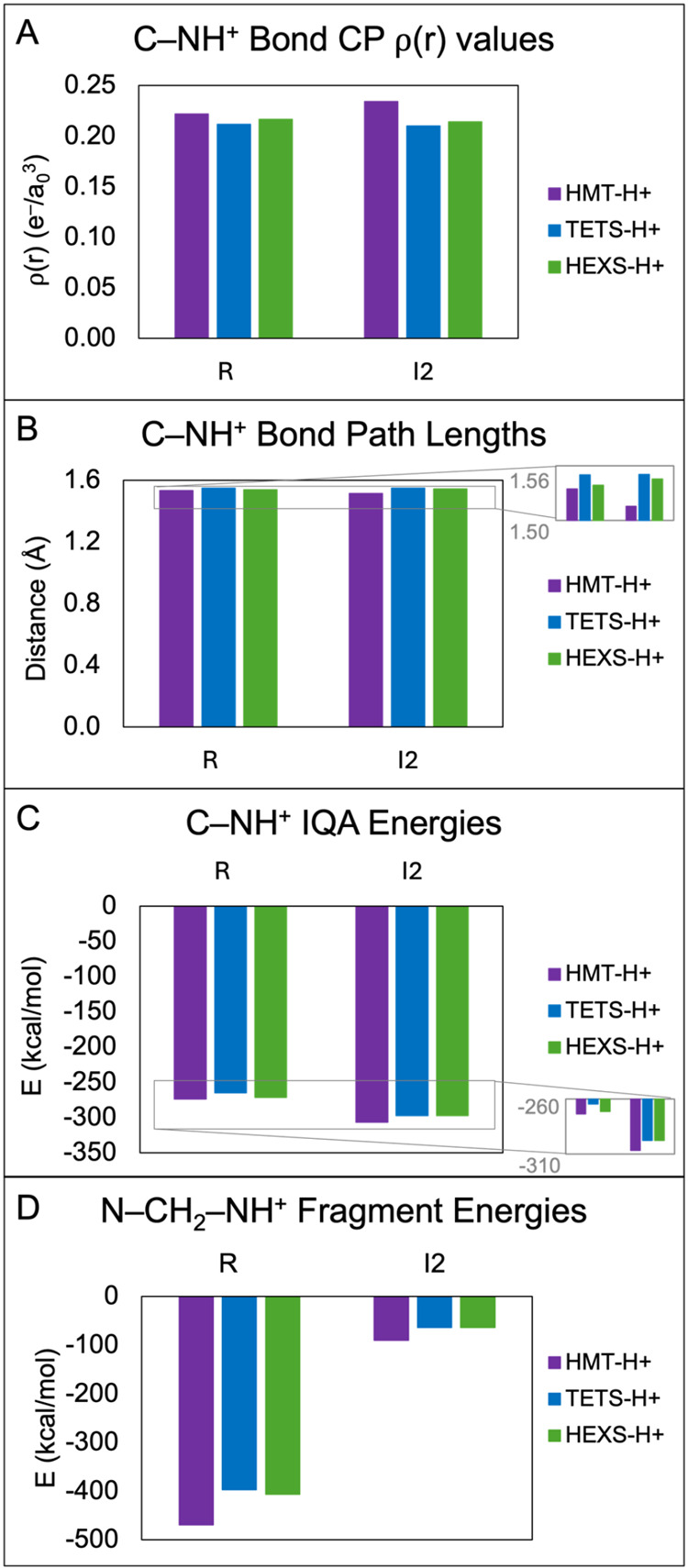
Analysis of stability of C–NH^+^ bonds in steps 1 and 3 of solvated reaction pathways for HMT, TETS, and HEXS. (A) Bond CP ρ­(*r*) values for the C–NH^+^ bond, (B) C–NH^+^ bond path lengths, (C) C–NH^+^ IQA energies, and (D) fragment BEs between the methylene group (CH_2_) and the rest of the molecule.

Comparing the three molecules tested here, the QTAIM results show that the HMT C–NH^+^ bonds were the most stable in every case, in alignment with activation barriers. For all cases with QTAIM, HEXS was more stable than (or similar to) TETS. For IQA energies, HMT was again the most stable in all cases. The C–NH^+^ bond in TETS was the least stable in both the reactant and intermediate compound using IQA analysis. Gas phase calculations gave similar results (Figure S10). For fragment energies, HMT was significantly more stable than HEXS and TETS in both the reactant and intermediate states. Overall, these data suggest that the C-NH^+^ bonds are most stable in HMT with TETS and HEXS showing lower stability. To further elucidate potential relationships between C-NH^+^ stability and heteroadamantane structure, we investigated a broader range of compounds to see if trends in C–NH^+^ bond stabilities emerged.

Figure S12 compares reactant stabilities for a set of seven protonated heteroadamantane compounds with structures for the four additional compounds shown in Figure S11. While there was a weak trend of decreasing stability as the compound size and number of sulfonyl groups increased, there were many exceptions. This may be at least partially due to the orientation of sulfonyl groups in the molecules. All three sulfonyl groups in HEXS are in the same plane, while the two sulfonyl groups in TETS are perpendicular to one another (see Figure S13). Overall, a clear correlation between the structure of heteroadamantanes and stability of C–N bonds was not determined.

For step 2 of the condensed-phase pathway, charge rearrangement occurred as protons were shuttled. The nitrogen atoms in I_1_ for TETS and HEXS were found to have more negative atomic charges than the nitrogen atom in HMT (charges and atom labeling shown in Table S9 and Figure S13). The more negative charges in TETS and HEXS likely led to the lower activation barriers calculated for step 2 (8.0 and 14.8 kcal/mol for TETS and HEXS versus 32.6 kcal/mol for HMT), as the nitrogen atom abstracts a proton from a water molecule. This charge rearrangement was facilitated by the presence of electron-withdrawing sulfonyl groups in TETS and HEXS, which are highly polarizable, allowing for easier charge transfer in neighboring atoms.

## Conclusions

This work sought to determine the unique properties of TETS and HEXS that led to the observed [M + H – CH_2_]^−^ precursor ions from (−)­ESI and to determine the plausibility of an in-droplet mechanism from acidic medium. Using DFT calculations, it was determined that protonation of a nitrogen atom in TETS, HEXS, and HMT weakened the C–NH^+^ bonds and could facilitate fragmentation within the ESI source. Considering the most favorable condensed phase pathways calculated, TETS and HEXS had considerably lower activation barriers than HMT for the formation of [M + H – CH_2_]^−^ starting from the relevant protonated compounds. Furthermore, it was determined that the mechanisms for TETS and HEXS could be achieved in the lifetime of a microdroplet formed in ESI, while in-source fragmentation of HMT would be less likely to occur.

In line with mechanistic results, QTAIM, IQA, and fragment energy analyses generally indicated the C–NH^+^ bonds in HMT to be more stable than those in TETS and HEXS. Using four additional heteroadamantane compounds, only a weak trend was established between increased molecule size and number of sulfonyl groups with weaker C–NH^+^ bonds, with HEXS often being an exception. However, the two sulfonyl groups in TETS and the three sulfonyl groups in HEXS still appear to be the major factors in destabilizing the C–NH^+^ bonds and leading to the observed loss of a methylene group during ESI.

Experiments were performed to determine if certain instrument parameters would affect the formation of HEXS from TETS as well as the [M + H – CH_2_]^−^ ions from both TETS and HEXS during (−)­ESI. When GC/MS analysis was performed on TETS in acetone or with formic acid, no presence of HEXS was detected. However, as seen in previous work, [HEXS + H – CH_2_]^−^ was observed in LC-(−)­ESI-MS/MS experiments, suggesting that the microdroplet environment or sufficient time exposed to acidic conditions and heat is necessary for the pseudodimerization of TETS to HEXS with the experimental conditions used in this work. Furthermore, [HMT + H – CH_2_]^−^ was not observed in LC-(−)­ESI-MS/MS experiments, in agreement with predictions from DFT calculations. There was a significant difference in the [HEXS + H – CH_2_]^−^ peak area compared to the [TETS + H – CH_2_]^−^ peak area when a capillary voltage of −4.5 kV was applied, which may have been caused by corona discharge at higher needle voltage settings or the larger molecular volume of HEXS compared to TETS. Based on calculated activation barriers, microdroplet lifetime during ESI, and the effect of capillary voltage, drying gas, and nebulizing gas on [M + H – CH_2_]^−^ peak areas, it seems likely that ionization of TETS and HEXS occurs in-droplet rather than as gas phase collision-induced dissociation and that careful optimization of ESI settings, including capillary needle voltage, is warranted for optimizing reaction efficiencies or minimizing unwanted byproducts. With all parameters tested in this work, the expected [M – H]^−^ precursor ions were never obtained with (−)­ESI from TETS. This work highlights the challenges in elucidating the origins of in-source fragmentation and reducing its impact in experiment.

## Supplementary Material



## Data Availability

All DFT calculation files can be found online: https://zenodo.org/records/18853042.

## Notes

GC/MS data, LC/MS and LC/MS/MS chromatograms for TETS, HEXS, and HMT, ^1^H NMR spectra, functional and basis set benchmark, proposed fragmentation pathway, LC/MS/MS Box-Behnken experiments, alternative mechanistic steps considered, gas phase reaction pathway, gas phase C–NH^+^ stabilities, fragment energies, stability trends for additional molecules, and atomic charges. (PDF)
