# Supplementary material for: DFT and Mass Spectrometry Study of (−)ESI-Induced Fragmentation of the Highly Toxic Rodenticide Tetramethylenedisulfotetramine
Source: ACS Omega. 2026 May 8;11(19):28582–91. doi: 10.1021/acsomega.6c00837 (PMC13191712; doi:10.1021/acsomega.6c00837)
Supplement: Supplementary file 1 [file ao6c00837_si_001.pdf]

Supplementary Information

DFT and Mass Spectrometry Study of (–)ESI-Induced Fragmentation of the Highly Toxic Rodenticide Tetramethylenedisulfotetramine

*Jonathan L. Gertner, Jacob Cook, Brett Mayer, Janel E. Owens, Amanda Morgenstern**

Department of Chemistry & Biochemistry, University of Colorado Colorado Springs, Colorado Springs, CO 80918

*Corresponding author: amorgens@uccs.edu

Table of Contents

1. GC/MS data
2. LC/MS and LC/MS/MS chromatograms for TETS, HEXS, and HMT
3. H-NMR Spectra
4. Functional and basis set benchmark
5. Proposed fragmentation pathway
6. LC/MS/MS Box-Behnken Experiment Results
7. Detection of proposed intermediates
8. Alternative mechanistic steps considered
9. Gas phase reaction pathway
10. Gas phase C-NH^+ stabilities
11. Fragment energies
12. Stability trends for additional molecules
13. Atomic charge analysis
14. References

1. GC/MS Data

A pure standard of tetramethylenedisulfotetramine (TETS) was prepared in GC-grade acetone with a concentration of 48 ng/µL. One µL injection onto a DB-5ms column on a 6890GC coupled with a 5973N MSD as described in the manuscript main text was completed to verify that the solution was pure and contained no HEXS (*m/z* 360) as shown in **Figure S1**. The chromatogram showed one main peak at 10.993 min (top panel) and the resulting full scan mass spectrum from *m/z* 50-450 (middle panel) confirmed the presence of TETS with the following ions: *m/z* 240, 212, and 121. The extracted ion chromatogram (bottom panel) showed that the ions at *m/z* 240 and 212 were the dominant ions for analysis and we saw no evidence of *m/z* 360 (red trace), which would indicate the presence of the dimer, hexamethylenedisulfotetramine (HEXS).

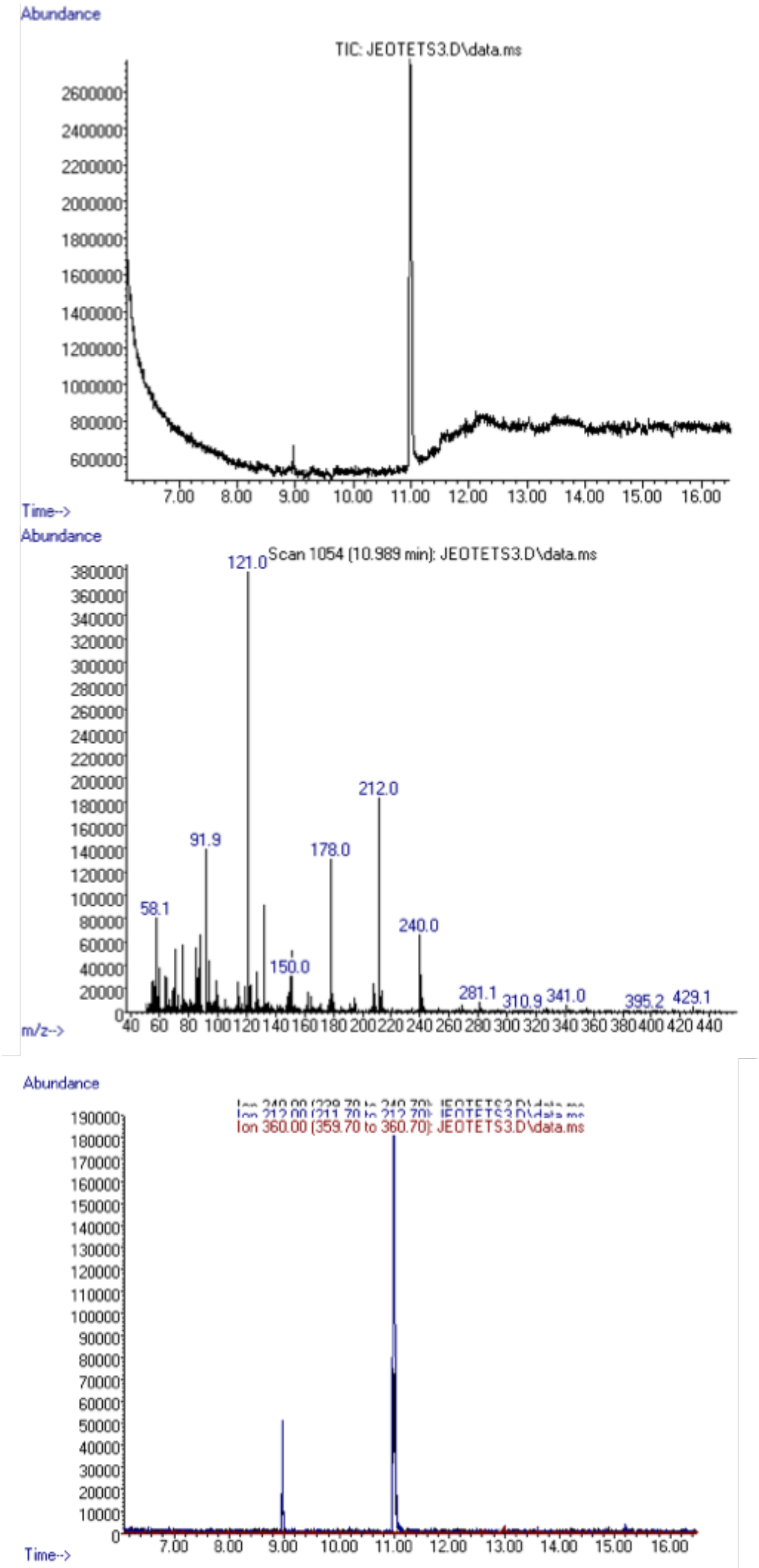

Figure S1. GC/MS results for TETS including chromatogram (top), mass spectrum (middle), and extracted ion chromatograms for ions *m/z* 240 and 212 (bottom), which indicate TETS, and *m/z* 360, which indicates HEXS (bottom).

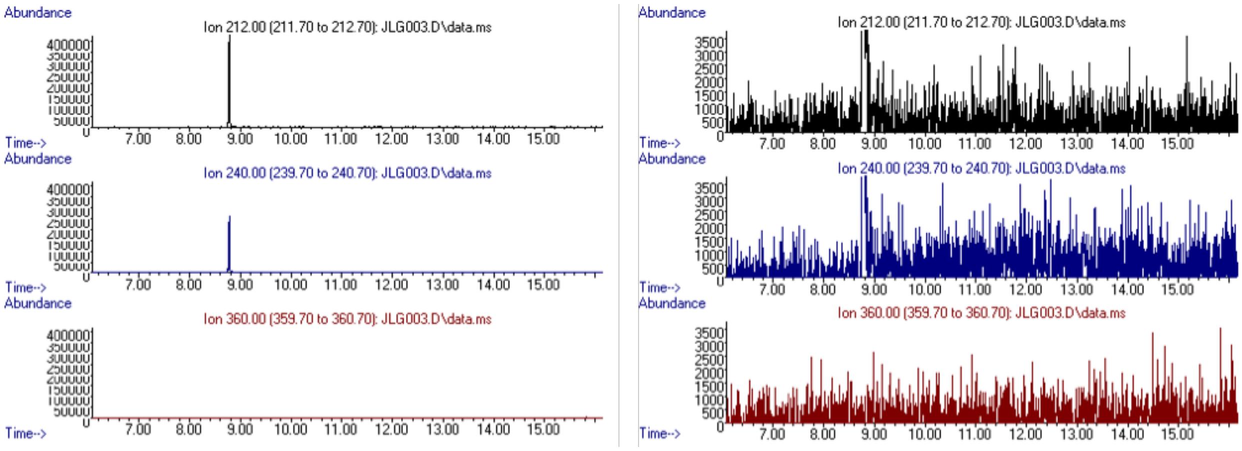

Figure S2. GC/MS results for the analysis of a TETS standard (20 ng/µL) stored in acetone and 0.1% formic acid for one week at room temperature (25 °C). Extracted ion chromatograms for *m/z* 212 (black trace) and *m/z* 240 (blue trace), which indicate TETS, and *m/z* 360 (red trace for HEXS) are shown in full scale (left panel) and zoomed in (right panel). We noted no formation of HEXS in this acidic environment at this ambient storage temperature. Note that the retention time for TETS here is different than **Figure S1** because a different temperature profile was used.

2. LC/MS Data

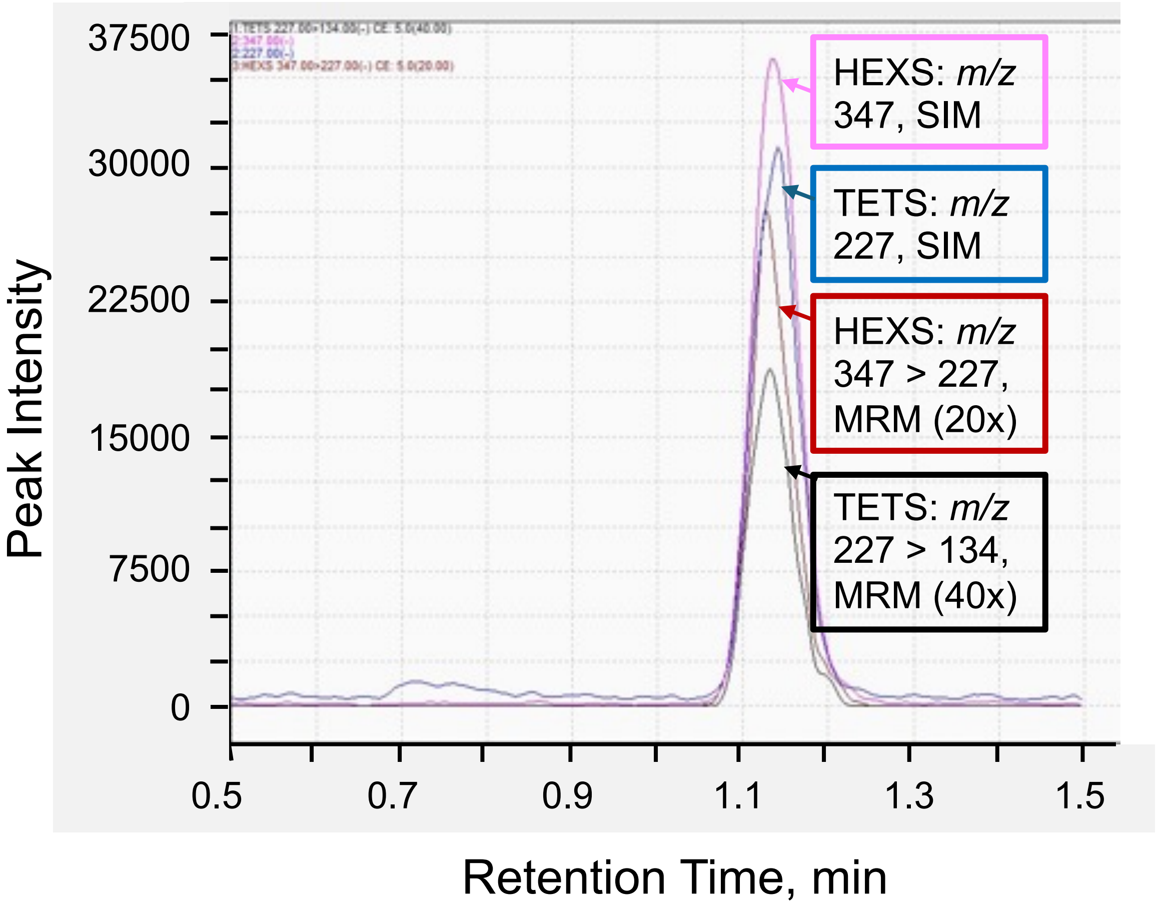

Figure S3. LC/MS (SIM) and LC/MS/MS (MRM) results for the analysis of an analytical standard of TETS (48 ng/µL). Here, the standard (2 µL injection) was analyzed at –1.5 kV capillary voltage, 11 L/min drying gas flow rate, and at 300 °C. The MRM traces for HEXS (*m/z* 347 > 227) and TETS (*m/z* 227 > 134) are increased by factors of 20 and 40, respectively, to aid in comparison of peak shape.

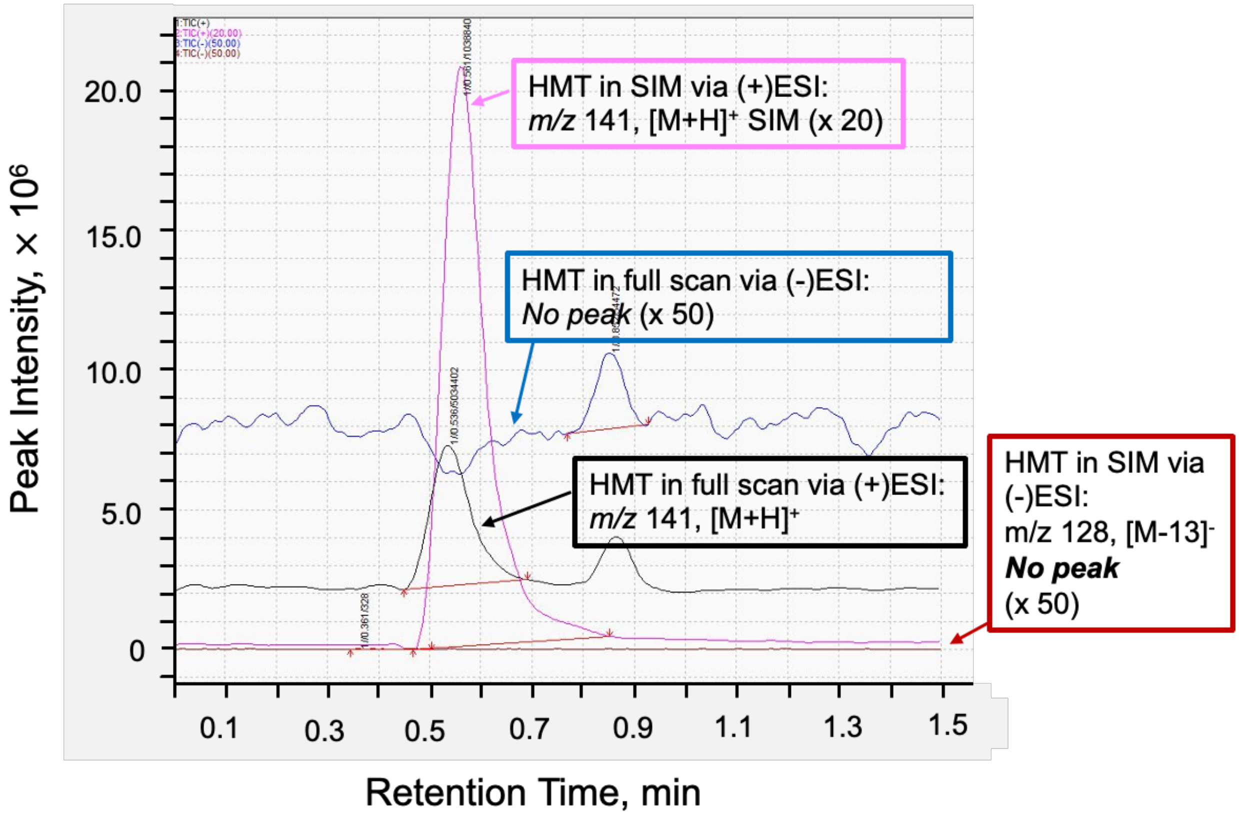

Figure S4. LC/MS (SIM) and full scan results for the analysis of an analytical standard of hexamethylenetetramine, HMT (2 ng/μL). Here, the standard (1 μL injection) was analyzed using +3.0 and –3.0 kV capillary voltages, 15 L/min drying gas flow rate, and with a heat block temperature of 300 °C. HMT was readily observable in (+)ESI, with the $[M + H]^+$ pseudo-molecular ion at *m/z* 141 observable in full scan (black trace) and SIM (pink trace with 20-fold signal enhancement) modes. HMT was **not observed** in (–)ESI (blue trace with 50-fold signal increase), and the loss of methylene bridge after protonation, or $[M + H^+ – CH_2]^–$, was not observed either (dark red trace with 50-fold signal increase).

3. NMR

H-NMR for TETS (**Figure S5**) and HMT (**Figure S6**) in deuterated acetonitrile with and without formic acid added. Also shown is acetonitrile and formic acid alone (**Figure S7**). Note the shoulder near the water peak around 2 ppm that occurs for TETS with formic acid in **Figure S5**. This peak does not occur in any of the other spectra and is likely an amine peak from a nitrogen atom in TETS being protonated from formic acid. The H-NMR data suggest that TETS is protonated in formic acid while HMT is not protonated.

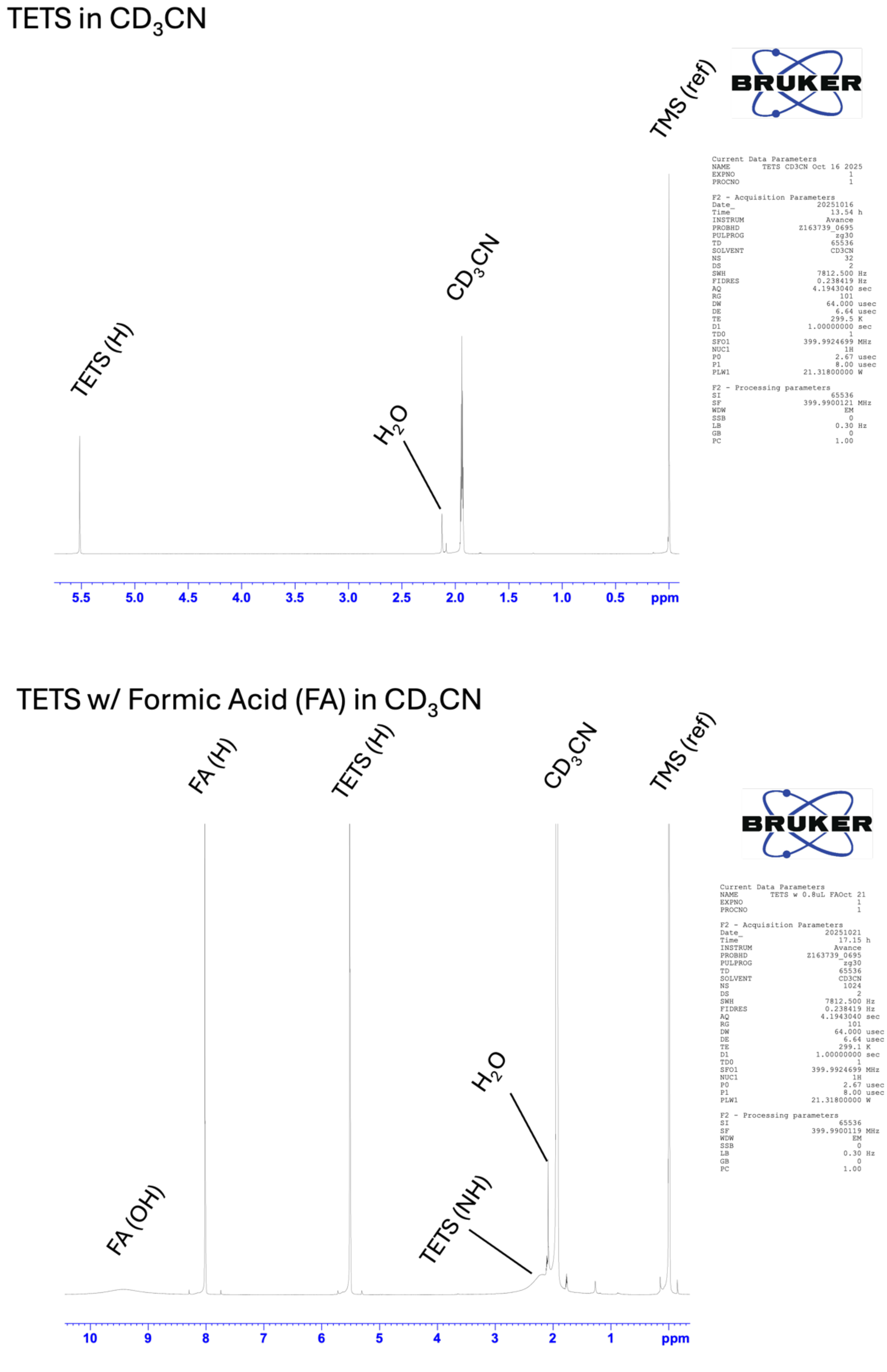

Figure S5. H-NMR of TETS in acetonitrile (top) and TETS with formic acid in acetonitrile (bottom).

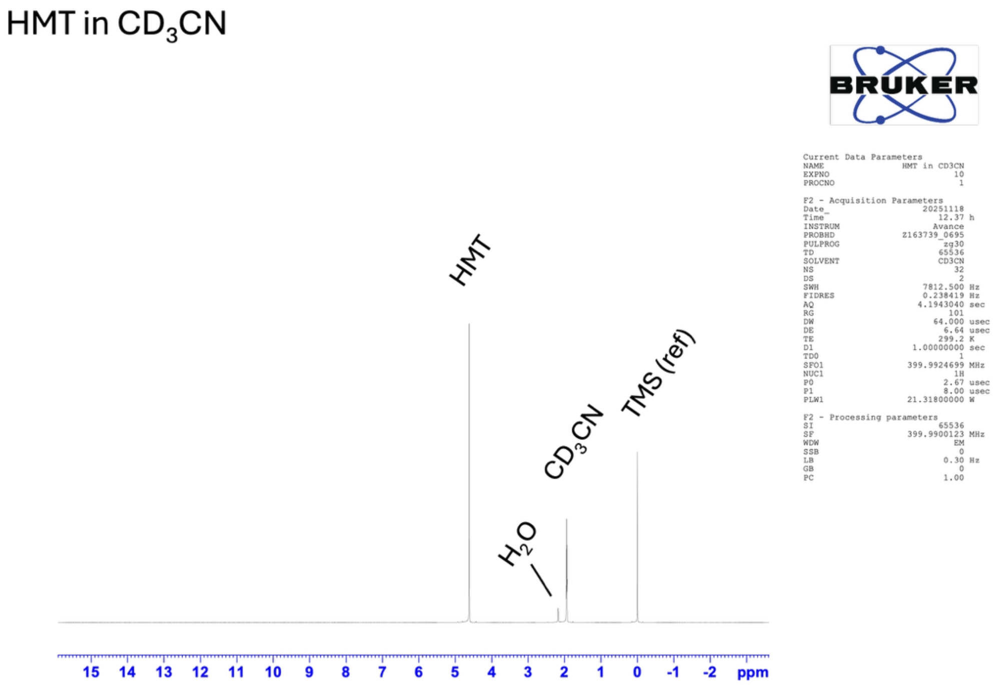

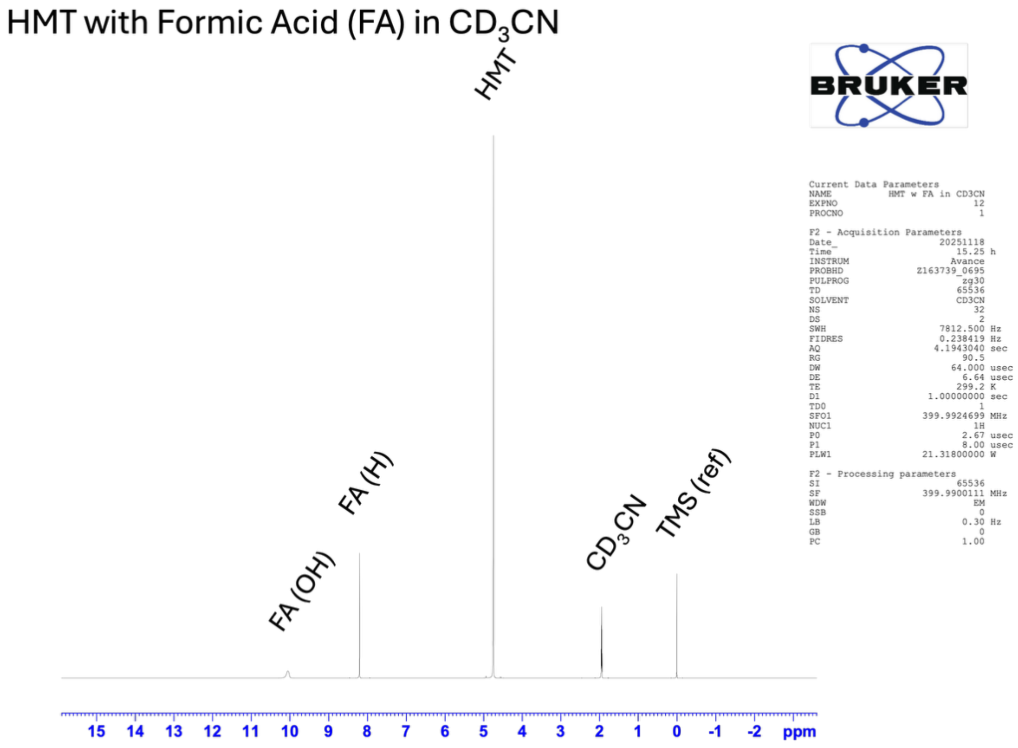

Figure S6. H-NMR of HMT in acetonitrile (top) and HMT with formic acid in acetonitrile (bottom).

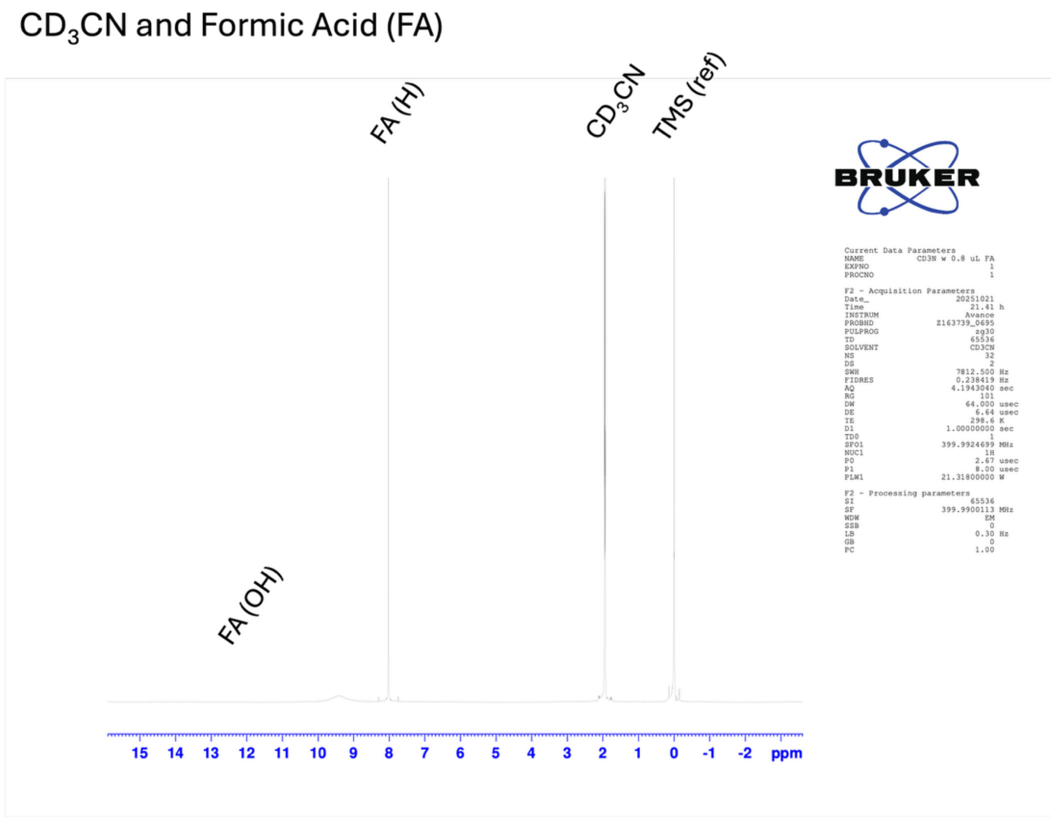

Figure S7. H-NMR of formic acid in acetonitrile.

4. Functional and Basis Set Benchmark

Table S1. Comparison of calculation methods (COSMO solvation with water or acetonitrile (MeCN), basis set/functional) for initial stability analysis of HMT and TETS. Small standard deviations exist between methods for CP ρ(r) and bond path length. While discrepancies for IQA energies occurred for different functional choices, B3LYP has been shown to accurately recover energies of systems with IQA methods. Thus, COSMO solvation with water and B3LYP/TZP were used for remaining calculations.

	**HMT**			**TETS**		
	IQA E (kcal/mol)	CP ρ(r) (e^-/a_0^3)	Bond path (Å)	IQA E (kcal/mol)	CP ρ(r) (e^-/a_0^3)	Bond path (Å)
H_2O, B3LYP/TZP	–297.03	0.2216	2.901	–267.74	0.2123	2.927
H_2O, PBE/TZ2P	–293.03	0.2222	2.901	–257.35	0.2155	2.925
MeCN, B3LYP/TZP	–297.09	0.2216	2.902	–267.72	0.2121	2.928
MeCN, PBE/TZ2P	–293.15	0.2215	2.902	–257.23	0.2153	2.926
Mean	-295.08	0.2217	2.902	–262.51	0.2138	2.927
Standard Deviation	2.29	0.0003	0.001	6.03	0.0019	0.001

5. Proposed fragmentation pathway

Scheme S1. Original proposed pathway for formation of $[M + H – CH_2]^{–}$ ions in LC-(–)ESI/MS/MS for HEXS, TETS, and HMT. Only a portion of the molecules are shown as depicted in **Figure 2** of the main text of the manuscript.

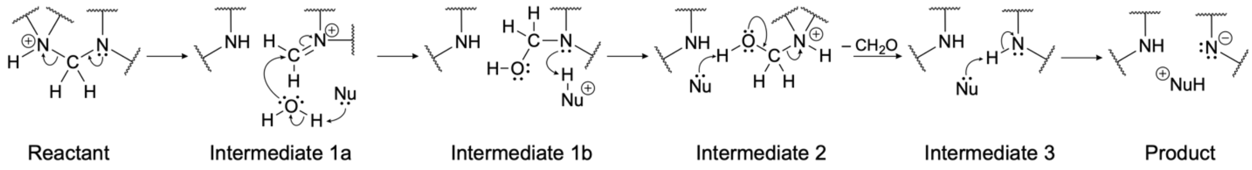

6. LC/MS/MS Box-Behnken Experiments

Table S2. Influence of ESI source conditions on ion formation as assessed by a Box-Behnken design for capillary voltage, drying gas flow rate, and heat block temperature (experiment 1). Single-factor ANOVA for the main effects of individual variables are shown at the top of the table and interactive effects are shown in the bottom half of the table.

	***P* Values for Main Effects (*F* value vs $F_{critical}$ = 3.219) with bolded values indicating statistical significance at *P* < 0.05**		
**SIM Analyses**	Capillary Voltage	Drying Gas Flow Rate	Heat Block Temp
***m/z* 347 (HEXS)**	**1.261×10^{-7}** (23.75)	**0.0068** (5.627)	**0.0147** (4.673)
***m/z* 227 (TETS)**	**5.847×10^{-6}** (16.27)	**0.0021** (7.145)	**0.0049** (6.041)
**Ratio of peak areas for HEXS/TETS**	**6.418×10^{-5}** (12.26)	0.5090 (0.686)	0.2153 (1.593)
**MRM Analyses**			
***m/z* 347 > 227 (HEXS)**	**1.536×10^{-6}** (18.72)	**0.0069** (5.612)	**0.0046** (6.127)
***m/z* 227 > 134 (TETS)**	**6.742×10^{-6}** (16.02)	**0.0018** (7.375)	**0.0068** (5.642)
**Ratio of peak areas for HEXS/TETS**	**0.0138** (4.744)	0.8972 (0.109)	0.0895 (2.558)
	***P* Values for Interactive Effects (*F* value vs $F_{critical}$ = 2.208) with bolded values indicating statistical significance at *P* < 0.05**		
**SIM Analyses**	Capillary Voltage × Drying Gas	Capillary Voltage × Heat Block	Drying Gas × Heat Block
***m/z* 347 (HEXS)**	**3.968×10^{-9}** (14.33)	**2.934×10^{-12}** (24.06)	**0.0025** (3.804)
***m/z* 227 (TETS)**	**1.010×10^{-7}** (11.07)	**6.093×10^{-12}** (22.89)	**0.0002** (5.333)
**Ratio of peak areas for HEXS/TETS**	**0.0025** (3.805)	**0.0005** (4.747)	0.0977 (1.859)
**MRM Analyses**			
***m/z* 347 > 227 (HEXS)**	**3.515×10^{-7}** (9.957)	**2.129×10^{-12}** (24.59)	**0.0006** (4.666)
***m/z* 227 > 134 (TETS)**	**2.336×10^{-7}** (10.31)	**6.188×10^{-12}** (22.87)	**0.0002** (5.420)
**Ratio of peak areas for HEXS/TETS**	0.1586 (1.602)	**0.0462** (2.250)	0.7741 (0.5966)

Table S3. Influence of ESI source conditions on ion formation as assessed by a Box-Behnken design for drying gas flow rate, nebulizing gas flow rate, and heat block temperature (experiment 2). Single-factor ANOVA for the main effects of individual variables are shown at the top of the table and interactive effects are shown in the bottom half of the table.

	***P* Values for Main Effects (*F* value vs $F_{critical}$ = 3.219) with bolded values indicating statistical significance at *P* < 0.05**		
**SIM Analyses**	Drying Gas Flow Rate	Nebulizing Gas Flow Rate*	Heat Block Temp
***m/z* 347 (HEXS)**	**0.0110** (5.030)	0.3189	0.0559 (3.091)
***m/z* 227 (TETS)**	**0.0005** (9.062)	0.2693	0.0510 (3.200)
**Ratio of peak areas for HEXS/TETS**	0.8296 (0.188)	0.1879	0.215 (1.596)
**MRM Analyses**			
***m/z* 347 > 227 (HEXS)**	**0.0121** (4.908)	0.2958	**0.0424** (3.410)
***m/z* 227 > 134 (TETS)**	**0.0124** (4.885)	0.3722	0.0524 (3.166)
**Ratio of peak areas for HEXS/TETS (MRM)**	0.9566 (0.0444)	0.0929	0.9741 (0.0262)
	***P* Values for Interactive Effects (*F* value vs $F_{critical}$ = 2.209) with bolded values indicating statistical significance at *P* < 0.05**		
**SIM Analyses**	Nebulizing Gas × Drying Gas	Nebulizing Gas × Heat Block	Drying Gas × Heat Block
***m/z* 347 (HEXS)**	**0.0004** (6.592)	$\mathbf{5.390 \times 10^{-8}}$ (11.66)	**0.0009** (4.408)
***m/z* 227 (TETS)**	**0.0004** (6.634)	$\mathbf{2.150 \times 10^{-8}}$ (12.56)	**0.0014** (4.144)
**Ratio of peak areas for HEXS/TETS**	0.3909 (1.085)	0.2495 (1.397)	**0.0189** (2.716)
**MRM Analyses**			
***m/z* 347 > 227 (HEXS)**	**0.0008** (5.895)	$\mathbf{7.596 \times 10^{-8}}$ (11.33)	**0.0010** (4.343)
***m/z* 227 > 134 (TETS)**	**0.0005** (6.395)	$\mathbf{2.630 \times 10^{-8}}$ (12.36)	**0.0011** (4.277)
**Ratio of peak areas for HEXS/TETS**	0.5743 (0.7778)	0.4474 (0.9809)	0.1213 (1.745)

*Reported *P* values were determined using Student's t-test because 0.5 L/min nebulizing gas produced no signal in most analyses. Comparisons in signal intensity for this main effect were between 1.5 L/min and 3.0 L/min settings, assuming H_0 = no difference between peak areas.

One unexpected result from this work is the significant difference in the relative formation of the HEXS ions (*m/z* 347 and product ions) versus TETS ions (*m/z* 227 and product ions) when varying the ESI voltage as displayed in **Table S2** considering that the calculated rates of reactions were similar for these two compounds. While this question is beyond the scope of the present work, it may be important to explore in future studies. ESI is complex in its three-step process: (1) nebulization of the liquid phase into charged droplets, (2) liberating the ionized analyte from those droplets, and (3) then transporting the ions from the ESI region into the mass spectrometer.[1] Because of the toxicity associated with TETS, the compound's mechanism of ionization via LC/MS/MS has not been widely studied, so its ionization energy or acidity/basicity characterization in liquid and gas phases are not know. Even less is known about its pseudo-dimer HEXS. The appearance of this HEXS ion (*m/z* 347) motivated the current study.

A review of the literature suggests that ionization efficiency varies within ESI, which leads to widely differing sensitivities of ESI-MS. Oss, et al., found that the most influential parameters in determining ionization efficiency of paired compounds were pK_a and molecular volume.[2] In their work, compounds that ionized via (+)ESI conditions to form $[M+H]^+$ pseudomolecular ions were studied, and there was an assumption that the concentrations of compound pairs would not affect ionization efficiency. They determined that compound size was an important parameter for stabilizing charge along with an increase in lipophilicity and decrease in basicity for these positive ions. This may be one reason why we see an increase in product ratio for HEXS vs. TETS under certain conditions.

Li, et al., explored vapor formation during droplet desolvation within ESI after citing important literature in their study that previously explored the composition of solvent on ionization efficiency.[3] In their work, they determined that ESI voltage, which was varied from 0

to 6 kV, had no effect on vapor velocity, which indicated that the vapor is mostly neutral and not accelerated by the applied potential field, even in the presence of analytes. The source of the vapor's velocity is attributed to the inertia of ESI droplets regardless of ESI source geometry.

These previous studies suggest that changing the voltage in ESI is not likely the sole reason for seeing changes in ionization ratios of TETS vs. HEXS, and it may motivate future areas of study.

7. Detection of Proposed Intermediates

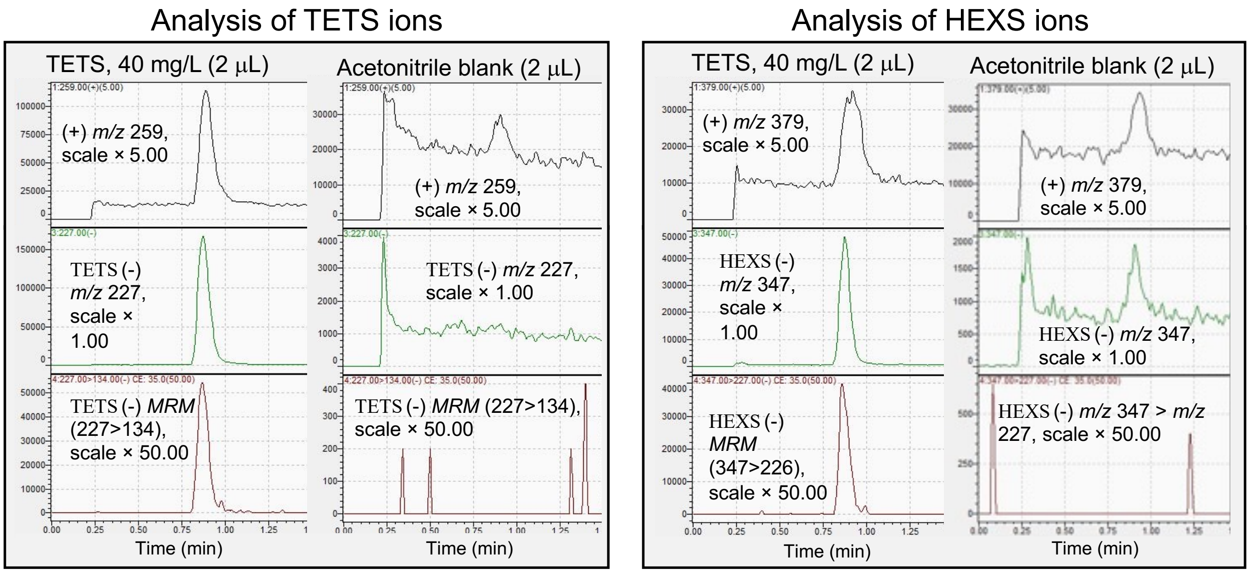

Figure S8. LC/MS (SIM) detection of $[M + 19]^+$ for TETS (left) and HEXS (right) corresponding to intermediate 1 and intermediate 2 in the proposed solvated pathway (**Figure 2A scheme a)** are shown in the top row. Note that intermediate 1 and intermediate 2 have identical chemical composition and correspond to *m/z* 259 in TETS and *m/z* 379 in HEXS and that these peaks are scaled × 5.00. For comparison, the middle row shows the observed precursor ions for TETS and HEXS (scale × 1.00) in SIM and the bottom row shows *m/z* 227 > 134 and *m/z* 347 > 226 for TETS and HEXS (scale × 50.00) in MRM. Acetonitrile blanks are shown to right of each TETS and HEXS spectra for background comparison.

The intermediates are all likely higher in energy than the reactant $[M + H]^+$ state, which we see by calculation, but we also found that the (–)ESI product, $[M + H – CH_2]^-$ was higher in energy than the intermediates (we refer the reader to **Figure 2** of the main text). However, we did not calculate the energy of the expected $[M + H ˆ CH_2]^+$ product ion from (+)ESI because we did not experimentally observe this ion.

8. Alternative Mechanistic Steps Considered

Scheme S2. Internal alcohol tautomerization mechanism through intermediate 2 hydronium proton transfer to the nitrogen. This scheme was only investigated for HMT and TETS as it was found to not be the most energetically favorable pathway for either of these two molecules.

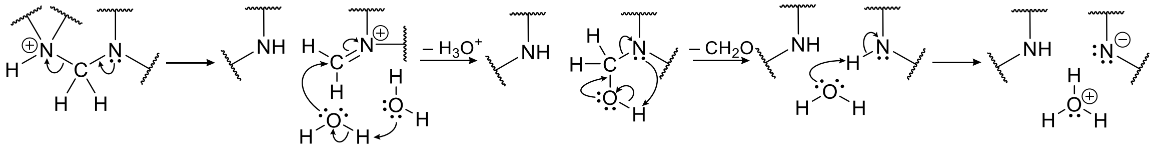

Table S4. – Condensed phase results of alternative pathways explored (room temperature).

Gibbs Free Energy (kcal/mol)	**Scheme S2**		
	HMT	TETS	HEXS
Reactant	–4081.20	–4200.02	–5919.72
TS1a	–4065.80	–4188.05	–5911.18
Intermediate 1a	–4073.76	–4196.66	–5918.41
TS1b	*	–4194.04	–5912.48
Intermediate 1b	–4040.60	–4195.32	–5915.53
TS2	–4015.67	–4186.44	–5900.56
Intermediate 2	–4035.86	–4195.33	–5911.88
TS3	–4035.90	–4181.98	–5898.29
Intermediate 3	–4038.58	–4188.72	–5909.93
Product	–3969.37	–4158.62	–5876.88

Gibbs Free Energy (kcal/mol)	**Scheme S3**	
	HMT	TETS
Reactant	–4081.20	–4200.02
TS1	–4065.80	–4188.05
Intermediate 1	–4073.76	–4196.66
TS2	*	–4194.04
Intermediate 2	–4040.60	–4195.32
TS3	–4015.67	–4152.11
Intermediate 3	–4038.58	–4191.06
Product	–3969.37	–4158.62

*Denotes a loose transition state where no transition state geometry was obtained

Table S5. – Gas phase results of alternative pathways explored (room temperature). Energy results for **Figure 2 – *Scheme b*** are shown in the main text for HMT.

Gibbs Free Energy (kcal/mol)	**Figure 2 – *Scheme b***	
	TETS	HEXS
Reactant	–4121.62	–5843.06
TS1	–4117.75	–5834.69
Intermediate 1	–4129.30	–5843.05
TS2	–4114.61	–5826.51
Intermediate 2	–4128.07	–5839.29
TS3	–4110.12	–5821.63
Intermediate 3	–4118.30	–5829.47
Product	–3927.58	–5658.28
Gibbs Free Energy (kcal/mol)	**Scheme S3**	
	HMT	TETS
Reactant	–4025.85	–4121.62
TS1	–4010.01	–4117.75
Intermediate 1	–4014.13	–4129.30
TS2	–3952.70	–4124.29
Intermediate 2	–3952.70	–4123.88
TS3	–3909.28	–4039.63
Intermediate 3	–3947.47	–4086.70
Product	–3741.09	–3927.58

9. Gas Phase Reaction Pathway

Gas phase pathway calculations were completed replicating all-electron B3LYP/TZP methods discussed in the main body of the text with two changes: no COSMO solvation was applied and the default room temperature (25 °C) was maintained. All reported transition states were found to have a single primary imaginary frequency, which was confirmed by follow-up IRC calculations to yield respective intermediate geometries. In some instances, additional low-magnitude imaginary frequencies (< -40 cm^{-1}) were observed to be associated with calculated geometries. Upon visualization, these were attributed to low-frequency motions of surrounding nonbonding molecules (i.e., water rotation). An additional round of geometry optimizations was subsequently performed, and these were found not to significantly affect the reaction pathway Gibbs energies or presence of the low-magnitude imaginary frequencies. One additional adjustment was applied during the acquisition of gas-phase TS_1 for HEXS wherein an internal harmonic restraint at a force constant of 0.35 a.u. was applied to hold the non-participating water molecules in their approximate location such that spatial consistency for the subsequent IM_2 geometry. This step was necessary due to the water molecules undergoing electrostatically driven migration toward the protonated nitrogen during the transition state search calculation, resulting in nonconvergence absent geometry restrictions. **Figure S9** shows the energy profile for the lowest energy gas phase pathway determined for HMT, HEXS, and TETS. The gas phase HMT pathway is identical to the condensed phase pathway for HMT shown in **Figure 2** of the main text. The gas phase HEXS and TETS pathway is shown in **Scheme S3**.

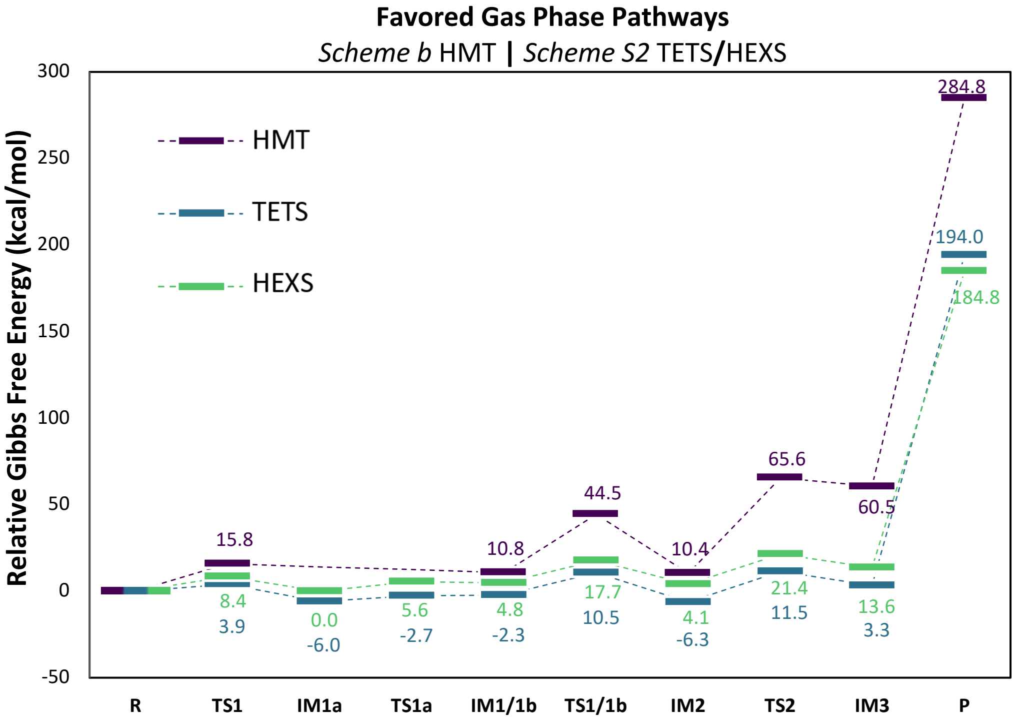

Figure S9. Energy diagram for lowest energy gas phase pathways found for formation of $[M + H - CH_2]^-$ ion in LC-(–)ESI/MS/MS for HEXS, TETS, and HMT at room temperature.

Scheme S3. Lowest energy gas phase pathway found for HEXS and TETS.

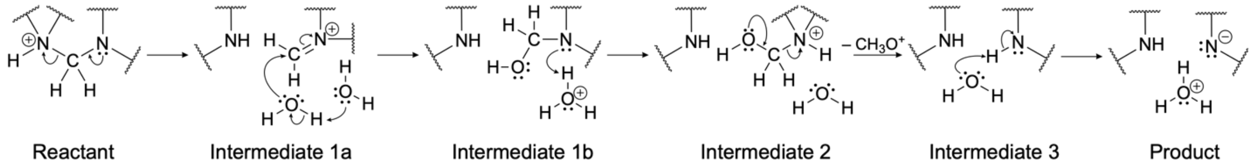

10. Gas phase C-NH$^+$ stabilities

There was not a large difference between the relative stabilities of reactant compounds when comparing the gas and condensed phases (**Figure 3** of the main text and **Figure S10**). Averaging across all three compounds and considering changes from the gas phase to the condensed phase, the amount of ρ(r) at bond CPs increased by 0.0045 e/a_0^3, bond path distances decreased by 0.010 Å, IQA energies decreased by 5.65 kcal/mol, and fragment energies increased by 320 kcal/mol.

For I_2 (step 3) there were a few instances of meaningful differences in C–NH$^+$ stability when comparing the gas and condensed phases. The only significant change in bond CP ρ(r) due to solvation was a decrease of 0.0156 e/a_0^3 in HMT. Similarly, the bond path of HMT increased by 0.030 Å and the IQA energy increased by 14.9 kcal/mol due to solvation, indicating lower C–NH$^+$ stability for HMT when solvated. TETS and HEXS had minor changes in bond path lengths and IQA energies. However, the fragment energies of all three compounds increased significantly when solvation was added, with an average increase of 43.2 kcal/mol. The large difference in fragment energies was likely due to the charges that were assigned to the fragments in each step. For step 1, the methylene group was assigned a +2 charge while a –1 charge was on the large "rest" of the molecule. For step 3, the protonated formaldehyde was positively charged while the "rest" of the molecule was neutral. Small, charged molecules, such as protonated formaldehyde or CH_2^{+2}, are generally less stable in the gas phase than a large, charged molecule (see **Table S6** for individual fragment energies).

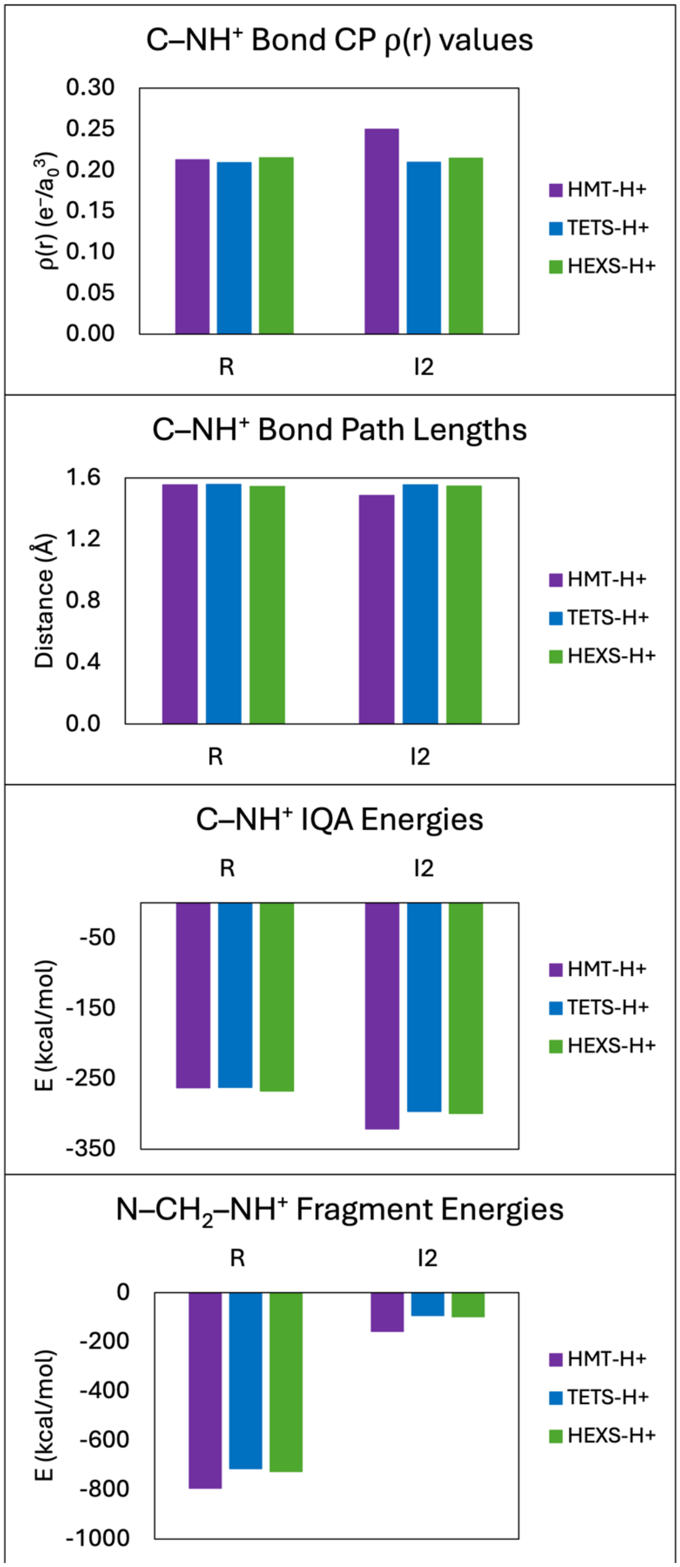

Figure S10. Analysis of stability of C–NH$^+$ bonds in steps 1 and 3 of gas phase reaction pathways for HMT, TETS, and HEXS. A) Bond CP ρ(r) values for C–NH$^+$ bond, B) N-CH_2–NH$^+$ bond path lengths, C) C–NH$^+$ IQA energies, and D) Fragment BEs between the methylene group (CH_2) and the rest of the molecule.

11. Fragment Energies

Table S6. Individual fragment bonding energies (BE) for HMT-H^+, TETS-H^+, and HEXS-H^+ in both the gas and condensed phases for reactant and intermediate 2 structures.

BE (kcal/mol)	HMT	TETS	HEXS
**Condensed Phase**			
Reactant – $rest^-$	–3096.10	–3252.30	–5012.83
Reactant – CH_2^{+2}	147.25	147.17	148.68
I_2 – rest	–3170.31	–3274.72	–5030.89
I_2 – CH_2OH^+	–427.40	–466.16	–464.93
**Gas Phase**			
Reactant – $rest^-$	–3033.09	-3188.78	-4946.21
Reactant – CH_2^{+2}	469.84	468.72	470.74
I_2 – rest	–3176.07	–3294.91	–5059.78
I_2 – CH_2OH^+	–537.45	–542.89	–545.48

12. Stability trends for additional molecules

In addition to HMT, TETS, and HEXS, the C–NH$^+$ bonds in the four molecules shown in **Figure S11** were used to examine stability trends in adamantane compounds with sulfonyl groups. MonoSO$_2$-1C ($C_5H_{10}N_4O_2S$) can be formed from ammonia, formaldehyde, and sulfamide. Reacting monoSO$_2$-1C with acid can give either diSO$_2$-1C ($C_7H_{14}N_6O_4S_2$) or HEXS. MonoSO$_2$-2C ($C_6H_{12}N_4O_2S$) has a second methylene linker between the two N atoms furthest from the sulfonyl group in monoSO$_2$-1C. While reaction of monoSO$_2$-2C was previously not found to yield diSO$_2$-2C ($C_8H_{16}N_6O_4S_2$), both compounds were still included in this computational study in an attempt to determine the effect of molecular weight, linker length, and number of sulfonyl groups on methylene stability.

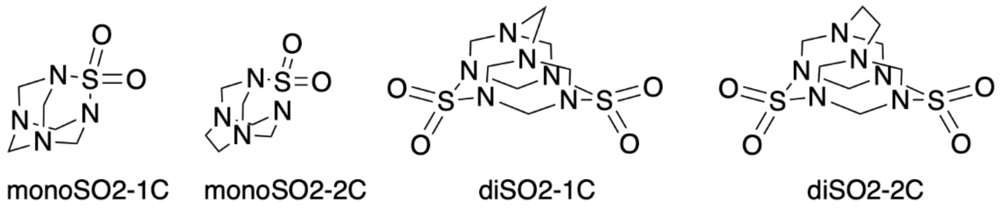

Figure S11. Structures of additional molecules included in C–NH$^+$ stability study

As these four molecules are less symmetric than the molecules included in the main text, multiple N protonation sites exist, and multiple subsequent methylene groups could be lost during ESI. Specifically, there are two unique N atoms that can be protonated in each molecule and either four (diSO$_2$ compounds) or three (monoSO$_2$ compounds) unique methylene groups that could be subsequently lost during ESI. The most likely N atom to be protonated on each molecule was first determined by protonating all symmetry unique nitrogen atoms. The total bonding energy (BE) of the entire molecule was then used to determine the **most stable** protonation site (**bolded** in **Table S7**).

Table S7. Bonding energies for unique protonation sites (**bolded** values are most stable).

Molecule	BE – N adjacent to SO$_2$ (kcal/mol)	BE – N away from SO$_2$ (kcal/mol)
monoSO2-1C	–3459.49	**–3468.42**
monoSO2-2C	–3882.21	**–3889.70**
diSO2-1C	–5221.03	**–5233.17**
diSO2-2C	–5635.85	**–5642.25**

From the protonation results, the ion with the lowest energy was then used to test the stability of methylene groups neighboring the protonated N atom. Three metrics were used to

determine stability: IQA energy, fragment energy, and bond CP ρ(r) values for C–NH+ bond. Results were mixed depending on the metric. For IQA energies, the methylene groups toward the C were least stable in all four molecules. For fragment energies, the methylene groups toward the sulfonyl group were less stable. For CP ρ(r) values, results were mixed depending on the molecule in question. IQA energies were ultimately used to choose the methylene group most likely to fragment. This metric was chosen as IQA energy looks at specific C-N bonding interactions while fragment energies look at interactions between compounds as a whole. The least stable methylene groups are **bolded** in **Table S8**.

Table S8. Energy analysis for unique methylene groups (**bolded** values are least stable).

	IQA Energy – CH_2 toward SO_2 (kcal/mol)	IQA Energy – CH_2 toward 1C or 2C (kcal/mol)	Fragment Energy – CH_2 toward SO_2 (kcal/mol)	Fragment E (CH_2 toward 1C or 2C (kcal/mol)	CP ρ(r) – CH_2 toward SO_2 (e^-/a_0^3)	CP ρ(r) – CH_2 toward 1C or 2C (e^-/a_0^3)
monoSO_2-1C	–272.13	**–269.65**	**–423.90**	–448.37	0.2259	**0.2196**
monoSO_2-2C	–263.81	**–240.30**	**–427.28**	–495.58	**0.2161**	0.2289
diSO_2-1C	–271.42	**–249.16**	**–428.08**	–446.71	0.2243	**0.1932**
diSO_2-2C	–260.72	**–233.66**	**–417.84**	–486.86	**0.2101**	0.2192

Stabilities of reactant C–NH^+ bonds for all seven molecules are shown in **Figure S12**. From the QTAIM analysis (**Figure S12**, Panels A and B), there was a mostly decreasing trend for stability with the addition of SO_2 groups and increasing size of compounds from HMT to diSO_2-1C. However, diSO_2-2C and HEXS both had increased values of charge density and decreased bond path lengths indicating more stable C–N bonds than the diSO_2-1C compound. For IQA energies (**Figure S12**, Panel C), a similar trend was found with C–N bonds generally becoming less stable with increasing size and number of SO_2 groups. However, here diSO_2-2C continued the trend but monoSO_2-2C was an outlier, demonstrating less stability than was anticipated.

The fragment results (**Figure S12**, Panel D) vary from QTAIM and IQA. This was reasonable as the fragment calculation provided an energetic stability of the entire methylene group rather than a single C–N bond. In this case, monoSO_2-2C, and diSO_2-2C had the least stable methylene groups and TETS, diSO_2-1C, and HEXS appeared to have the most stable methylene group. HMT and monoSO_2-1C have intermediate energies. Because both C–N bonds do not break at the same time, this analysis method was likely least applicable for determining which compounds were most likely to undergo cleavage during ESI.

There was a general trend of decreasing stability as the compound size and number of sulfonyl groups increased, except for HEXS in all cases and the additional exception of diSO2-2C with QTAIM analyses. HEXS was also more stable than anticipated. Due to these multiple exceptions, a general trend between number of sulfonyl groups, molecular size, and stability of C–N bonds could not be determined.

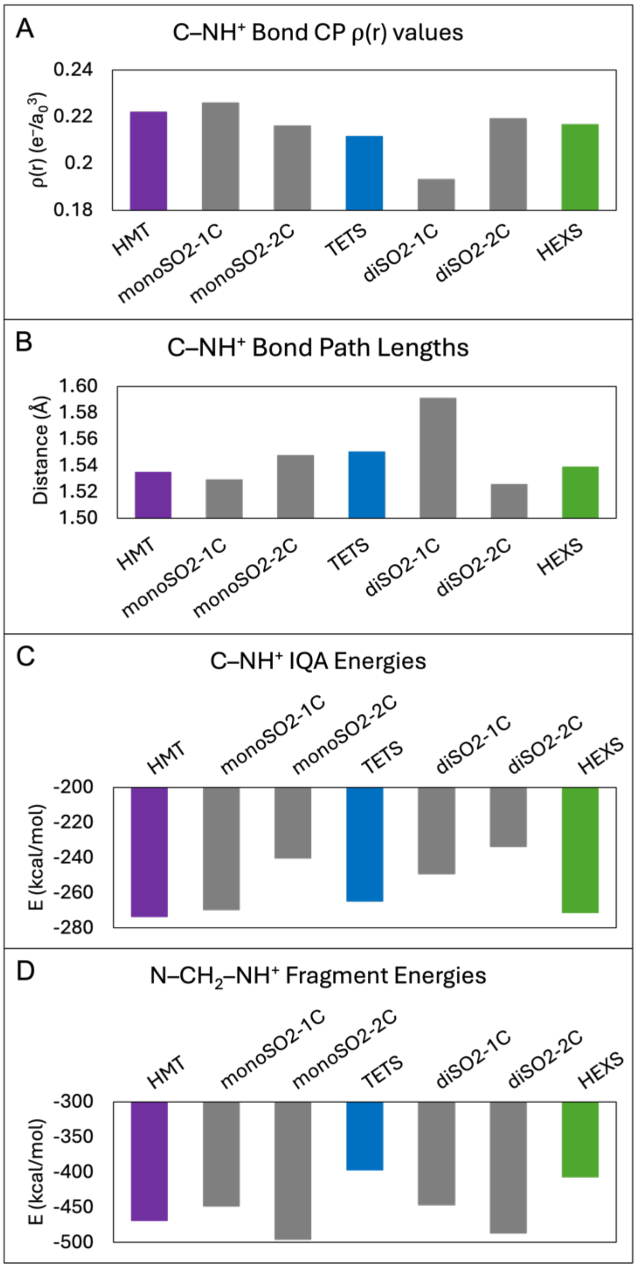

Figure S12. Stabilities of methylene groups in seven protonated heteroadamantane structures with COSMO solvation. A) bond CP r(r) values for C–NH$^+$ bond, B) C–NH$^+$ bond path length, C) C–NH$^+$ IQA bond energies, and D) Fragment energies between the methylene group (CH_2) and the rest of the molecule. Note that *y*-axes have been adjusted to emphasize differences between molecules

13. Atomic charge analysis

Table S9. Bader atomic charges calculated with QTAIM for intermediate 1 (I_1), intermediate 1a (I_{1a}), and intermediate 1b (I_{1b}). Atoms corresponding to the atomic charges are highlighted in **Figure S13.**

	C	N	O	SO_2	CH_2*
**Condensed Phase**					
HMT (I_1)	0.6657	–0.9734	n/a	n/a	0.7179
TETS (I_1)	0.7199	–1.0483	–1.0292	0.5336	n/a
HEXS (I_1)	0.7355	–1.0807	–1.0315	0.6141	n/a
**Gas Phase**					
HMT (I_1)	0.6809	–0.9553	n/a	n/a	0.6866
TETS (I_{1a})	0.688	–1.0163	n/a	0.6549	n/a
HEXS (I_{1a})	0.6938	–1.0132	n/a	0.6978	n/a
TETS (I_{1b})	0.7399	–1.0818	–1.0269	0.5784	n/a
HEXS (I_{1b})	0.7523	–1.0565	–1.0479	0.6533	n/a

*Average of two CH_2 groups

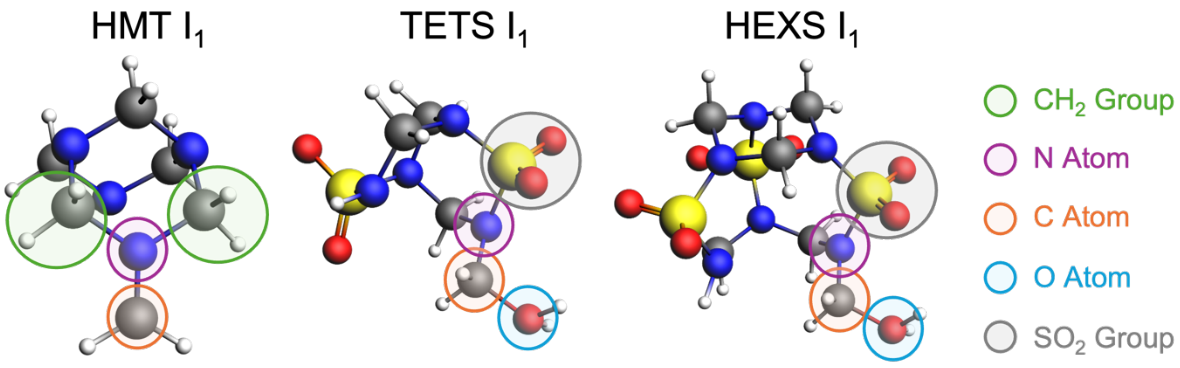

Figure S13. Structures of Intermediate 1 for HMT, TETS, and HEXS in the condensed phase. Atoms and groups with Bader charges shown in **Table S9** are highlighted.

14. References

1. Bruins, A. P. "Mechanistic aspects of electrospray ionization." *J. Chromatogr. A*, **1998**, *794*, 347-357. DOI: 10.1016/S0021-9673(97)01110-2. https://doi.org/10.1016/S0021-9673(97)01110-2
2. Oss, M.; Kruve, A.; Herodes, K.; Leito, I. "Electrospray ionization efficiency scale of organic compounds." *Anal. Chem.* **2010**, *82*, 2865-2872. https://doi.org/10.1021/ac902856t.
3. Li. H.-I.; Prabhu, G. R. D.; Buchowiecki, K.; Urban, P. L. "High speed Schlieren imaging of vapor formation in electrospray plume." *J. Amer. Soc. Mass Spectrom.* **2024**, *35*, 244-254. https://doi.org/10.1021/jasms.3c00345
